# Incorporating the direct derivation method and molecular scattering power method into the Rietveld quantitative phase analysis routine in *TOPAS*

**DOI:** 10.1107/S1600576725004054

**Published:** 2025-06-16

**Authors:** Xiaodong Wang, Henry Spratt

**Affiliations:** a Central Analytical Research Facility Queensland University of Technology (QUT) Level 6, P Block, Gardens Point Campus Brisbane Queensland 4001 Australia; Institut de Recherche sur les Céramiques, France

**Keywords:** direct derivation method, molecular scattering power, Rietveld, quantitative phase analysis, QPA, *TOPAS*

## Abstract

The equations of the direct derivation method (DDM) and the unit-cell scattering power method are reviewed in this report. Their relationships and connections to the conventional Rietveld quantitative phase analysis (QPA) are revealed, leading to the development of the *C_k_*-corrected DDM and the molecular scattering power (MSP) method. Both methods can be seamlessly integrated into the conventional Rietveld QPA routine as hybrid QPA, *i.e.* they enable fitting phases of partially or no known crystal structure simultaneously with conventional crystal structure modelling of other known crystalline phases. The accuracies of these hybrid QPA methods are evaluated using a calculated X-ray diffraction pattern for a mixture, the IUCr round robin CPD-1 dataset and synthetic mixtures of disordered source clay minerals (kaolinite KGa-2, chlorite CCa-2) with corundum, using both Launch Mode and Graphical User Interface (GUI) Mode of the *TOPAS* software. Although the accuracies of these hybrid QPA methods are slightly lower than that of conventional Rietveld QPA, their absolute deviations from weighed percentages are scarcely larger than 3 wt%. Compared with the original DDM, the *C_k_* correction enhances QPA accuracy, particularly for mixtures containing phases of large differences in average atomic number. An advantage over the original unit-cell scattering power method is that the proposed MSP method eliminates the need to know the lattice parameters, unit-cell volume or number of molecules in the unit cell.

## Abstract 2

Two recently reported quantitative phase analysis (QPA) methods—the direct derivation method (DDM) and the unit-cell scattering power method—have been further developed into the *C_k_*-corrected DDM and the molecular scattering power method, respectively. These methods are compatible with the conventional Rietveld QPA routine, as demonstrated through quantification of disordered clay mineral phases using the *TOPAS* software.

## 1. Introduction

Conventional Rietveld quantitative phase analysis (QPA) requires that the crystal structures of analytes are known (Hill & Howard, 1987 ▸; Bish & Howard, 1988 ▸). For QPA involving phases of partially or no known crystal structure, the PONKCS method (Scarlett & Madsen, 2006 ▸) can be employed. This method requires a preliminary experimental step to calibrate the *ZMV* factor—the product of unit-cell mass and unit-cell volume—of the PONKCS phase (Wang *et al.*, 2011 ▸). Alternatively, calibration can be achieved by refining the atomic occupancies of an uncertain structural model, such as the interlayer exchangeable cations or water content in swelling clay minerals (Wang *et al.*, 2012 ▸). These calibration procedures typically involve (1) enriching the unknown phase from the sample to be quantified, (2) preparing a standard mixture—usually in 1:1 weight ratio—of the enriched unknown phase and a well-characterized crystalline standard, and (3) scanning the standard mixture under the same instrument conditions used for all samples containing the unknown phase. Therefore, the application of the PONKCS method is limited to scenarios in which these experimental calibration steps can be performed.

The direct derivation method (DDM) (Toraya, 2016 ▸) is a relatively new QPA technique primarily developed for phases with unknown crystal structure, offering the distinct advantage of ease of use. DDM calculates the unknown phase’s scattering power per unit mass, denoted as 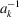, from its chemical composition (Toraya, 2017 ▸), thereby avoiding the laborious calibration steps required by methods such as PONKCS. As long as the chemical compositions for all the analytes are known, QPA can be performed using DDM via the whole-powder-pattern fitting procedure (Toraya, 2018 ▸).

**Figure 1:**
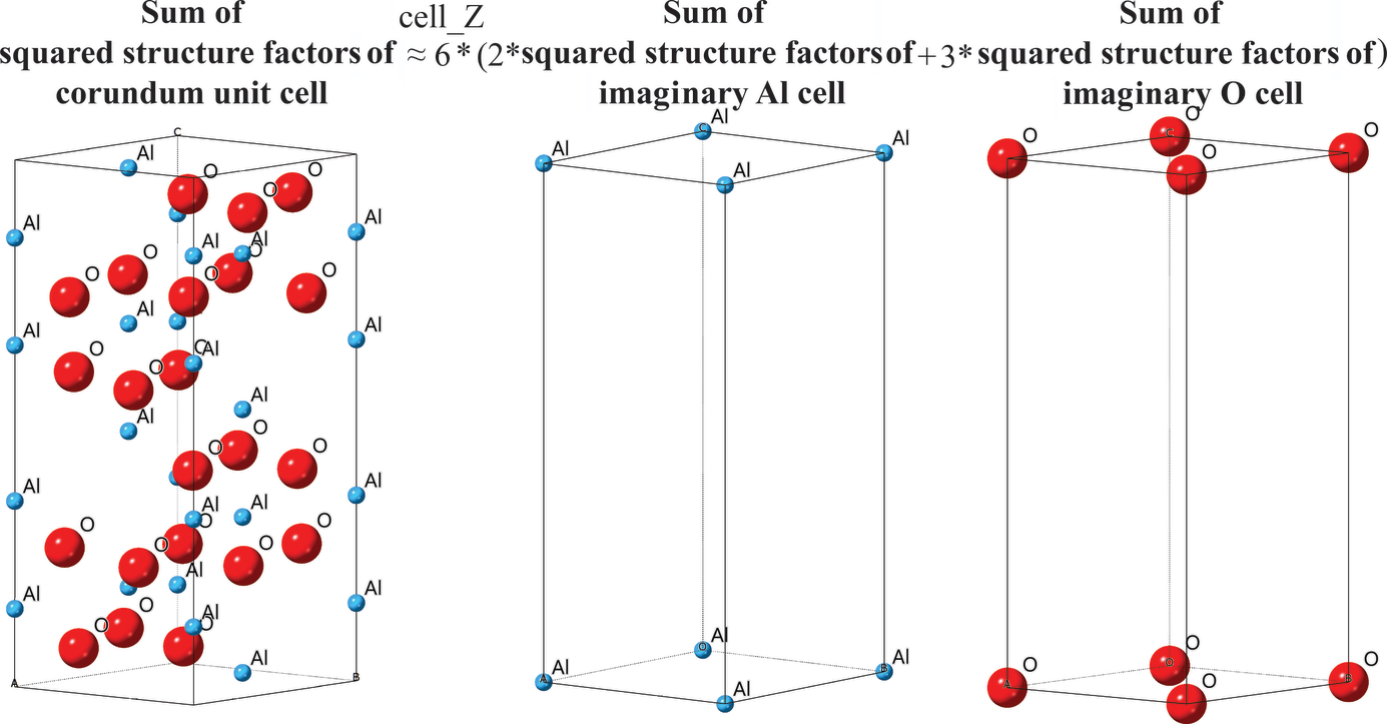
The application of the unit-cell scattering power method (Li *et al.*, 2022 ▸) on corundum (α-Al_2_O_3_). The sum of squared structure factors of a corundum cell can be estimated through the sum of squared structure factors of 12 imaginary Al crystals and 18 imaginary O crystals in the corundum unit cell. ‘cell_Z’ denotes the number of Al_2_O_3_ molecules in the corundum unit cell.

However, the theoretical foundation of DDM assumes that (*a*) the sum of squared structure factors for all reflections within an appropriately wide 2θ range can be approximated by (*b*) the product of unit-cell volume and the sum of squared electron numbers for all atoms in the unit cell—a fixed number for a particular phase (Toraya, 2016 ▸). Naturally, the validity of this assumption depends on the appropriateness of the chosen 2θ range. Although the ratios *C* between (*a*) and (*b*) have been plotted against the upper limit of the 2θ range (2θ^UL^) for 13 phases and deemed to be ‘close’ to each other [Fig. 1 ▸ of Toraya (2016 ▸)], counter-examples have been raised by He & Li (2022 ▸), in which the *C* ratios differ significantly among component phases over any 2θ range, leading to wrong QPA results from DDM that deviate more than 20% from those obtained using the conventional Rietveld QPA method.

Instead, Li *et al.* (2022 ▸) proposed a unit-cell scattering power method to estimate the sum of squared structure factors of an analyte by using the sum of squared structure factors of a series of imaginary crystals. Each of these imaginary crystals contains a single constituent atom in the analyte’s unit cell positioned at the origin of the same unit cell. An example for corundum is illustrated in Fig. 1 ▸.

This approach is based on the principle that the structure factor of a unit cell is the Fourier transform of its electron density. According to Parseval’s theorem (Pollard, 1926 ▸; Hughes, 1965 ▸; Zwillinger, 2014 ▸), the sum of squared structure factors equals the integral of squared electron density over the unit cell. If this integral can be approximated by the sum of the squared electron densities of individual atoms in the unit cell, *i.e.* ignoring electrons on interatomic bonds, then the sum of squared structure factors can also be approximated in this manner. This offers an alternative way to evaluate the total scattering power of phases with partially known structures, facilitating phase quantification without requiring knowledge of atomic positions. Unfortunately, the authors did not find suitable software to implement their method and hence devised equations for calculating relative intensity ratios (RIR), a non-refinement-based QPA method (Li & He, 2023 ▸).

The present report reviews the equations used in Rietveld QPA alongside those of DDM and the unit-cell scatting power method, leading to the development of a *C_k_*-corrected DDM and a molecular scattering power (MSP) method, respectively. Both methods are shown to be compatible with the conventional Rietveld QPA routine. Incorporating individual *C_k_* values for different phases improves the QPA accuracy of DDM. The MSP method simplifies analysis by eliminating the need to know the lattice parameters or unit-cell volume of the unknown crystalline phase. Examples are provided using the *TOPAS* v7 software (Coelho, 2018 ▸), including INP templates to calculate the *C_k_* ratio for any measured 2θ range, demonstrating Pawley fitting to fit only the unknown phase together with Rietveld refinements for known phases. Equations for *C_k_*-corrected DDM calculations and the MSP method are implemented in the Rietveld QPA routine of both Launch Mode and Graphical User Interface (GUI) Mode in the *TOPAS* v7 software. An INP template is also provided to calculate the MSP value for any chemical formula. Both methods are demonstrated to be equivalent to conventional Rietveld QPA, as theorized by the final equation in Section 2.

## 2. Theory

### 2.1. Rietveld QPA

The diffraction intensity of the *j*th reflection of the *k*th phase in a multi-phase mixture measured using Bragg–Brentano geometry can be written as (Madsen & Scarlett, 2008 ▸)
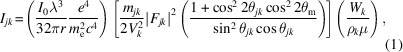
where *I*_0_ is the incident beam intensity, λ is the X-ray wavelength, *e* is the electron charge, *m*_e_ is the electron mass, *r* is the goniometer diameter, *c* is the speed of light, *m* and *F* are the multiplicity and structure factor, respectively, *V* is the unit-cell volume, and θ_*j*_ and *θ*_m_ are the Bragg angles for the *j*th sample reflection and for the monochromator, respectively. *W_k_* and *ρ_k_* are the weight fraction and density of phase *k*, while μ is the mass absorption coefficient of the mixture sample.

We use *Q* to represent the physical and geometrical constants 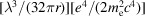 and 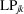 to represent 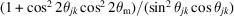, the Lorentz and polarization factor. Then substituting 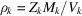 into equation (1) and summing over all 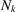 reflections gives
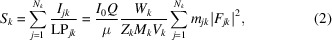
where *M_k_* is the molecular weight and *Z_k_* is the number of molecules in unit cell. For simplicity, the LP-factor-corrected diffraction intensity in equation (2) is denoted as *S_k_*. Re-arranging equation (2) provides the weight fraction of phase *k*:
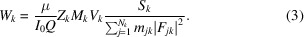


In Rietveld QPA, the last term of equation (3)—the ratio between the sum of LP-factor-corrected intensities 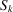 and the sum of structure factors—is refined as the Rietveld scale factor:
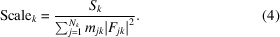


Among all *K* component crystalline phases in the mixture, the relative weight fraction of phase *k* can be derived as
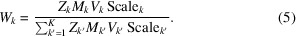


Equation (5) is widely used in many Rietveld software platforms that are currently publicly available for QPA and is hereafter referred to as ‘Rietveld QPA’.

### 2.2. *C_k_*-corrected direct derivation method

Toraya (2016 ▸) pointed out [equation (8) therein] that the scattering power of phase *k*, calculated from its crystal structure in Rietveld QPA as the sum of squared structure factors [*i.e.* the denominator of equation (4)], can be approximated using a formula involving the sum of squared electron numbers of each atom, which can be calculated directly from the chemical formula of phase *k*:
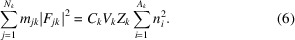


In the right-hand term of equation (6), *n_i_* is the electron number for the *i*th atom out of a total of *A_k_* atoms in the chemical formula (hereafter ‘molecule’), while *C_k_* is the ratio between these two forms of X-ray scattering power for phase *k*. With the above replacement, equation (3) can be written as
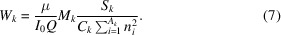


Toraya (2017 ▸) further grouped the parameters originating from the nature of the phase *k* as *a_k_*,
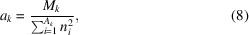
where the physical meaning of 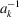 is the scattering intensity per unit mass of phase *k*. Therefore, the equivalent relative weight fraction of phase *k* in DDM follows equation (9),
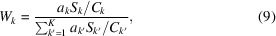
except that *C_k_* was assumed to be the same for all phases and hence got cancelled in the original DDM (Toraya, 2016 ▸). Comparing equation (9) and equation (5), it is easy to find the relationship between DDM and the conventional Rietveld QPA:
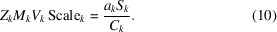


Equation (10) allows incorporation of a *C_k_*-corrected DDM calculation for phase *k* into the conventional Rietveld QPA routine, avoiding any experimental calibration step for the *ZMV* factor in the PONKCS method if the value of *C_k_* is known.

For poorly crystalline phases—such as those exhibiting severe structural disorder—conventional Rietveld refinement may not adequately fit the area under the diffraction peaks, from which QPA results are derived. In general, Pawley or Le Bail fitting (*TOPAS*hkl_Is model) and the peaks phase fitting (*TOPAS*xo_Is model) provide better fits to the peak areas and shapes of the whole powder pattern than the Rietveld method (*TOPAS*str model), because of their individual control of peak intensities, profiles and positions. With a *C_k_* value calculated from a sufficiently similar, disorder-free crystal structure of the same or similar phase (available from crystal structure databases), equation (9) enables quantification of disordered or poorly crystalline phases using hkl_Is models (Pawley or Le Bail fitting) or xo_Is models (whole-powder-pattern fitting), without the need to explicitly model disorder-induced peak profile changes.

### 2.3. Molecular scattering power method

Li *et al.* (2022 ▸) revisited equation (3) and proposed the unit-cell scattering power method. It approximates the sum of squared structure factors for the unit cell of phase *k* [the denominator of equation (4)] using the sum of squared structure factors from a series of imaginary crystals of the same unit cell, each with only a single constituent atom of phase *k* placed at their origins (as has been illustrated in Fig. 1 ▸):
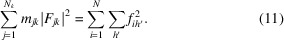


In the right-hand term of equation (11), 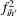 stands for the squared structure factor of the 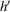 reflection from imaginary crystals consisting of only the *i*th atom—out of a total of *N* = *Z_k_A_k_* constituent atoms in the phase *k* unit cell—sitting at the origin of the unit cell of phase *k*. Although this unit-cell scattering power approach does not require atomic position information, it still relies on the known lattice parameters and the molecular motif of the target phase *k*.

However, if one studies the equations provided by Li *et al.* (2022 ▸), the requirement of ‘known lattice parameters’ is not necessary. Following the idea of Li *et al.* (2022 ▸), since the structure factor *F_hkl_* is the Fourier transform of the electron density distribution ρ(**r**) in the unit cell, according to Parseval’s theorem (Pollard, 1926 ▸; Hughes, 1965 ▸; Zwillinger, 2014 ▸), the sum of squared structure factors ∑|*F_hkl_|*^2^ equals the integral of the squared electron density distribution ρ(**r**) over the unit cell:^1^
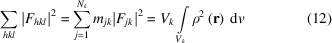


If the electron density distribution of the unit cell can be approximated by the sum of the electron density distributions of isolated atoms, *i.e.* ignoring the electron density charges on interatomic bonds, then the squared electron density distribution of the unit cell ρ^2^(**r**) can be approximated by the sum of squared electron density 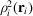 of each atom for all *Z_k_A_k_* atoms in the unit cell, or in a mathematical expression
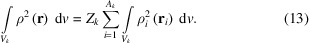


Considering the last integral in equation (13), the integration volume *V_k_* (unit-cell volume of phase *k*) is commonly much larger than the atomic volumes, outside of which *ρ_i_*(**r***_i_*) reaches zero. The integration result, therefore, does not depend on *V_k_* the region of integration, *i.e.* the following relation holds for the same atom:
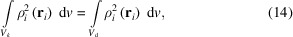
where *V*_d_ is any arbitrary dummy volume providing it is much larger than the atomic volumes. If we create *A_k_* imaginary crystals in arbitrary dummy unit cells (*e.g.* cubic cells of lattice parameter *a* = 10 Å) and place each constituent atom of the phase *k* molecule on their origins (space group *P*1, *x* = *y* = *z* = 0), the following relation holds for these imaginary crystals [*cf.* equations (12) and (13)]:
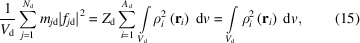
where *m*_*j*d_ and *f*_*j*d_ are the multiplicity and structure factor, respectively, of the *j*th reflections (*j* = 1 to *N*_d_) from the imaginary dummy crystals. Since there is only one atom in these dummy cells, *Z*_d_ = *A*_d_ = 1. Bringing equations (12)–(15) together we have
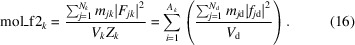


**Figure 2:**
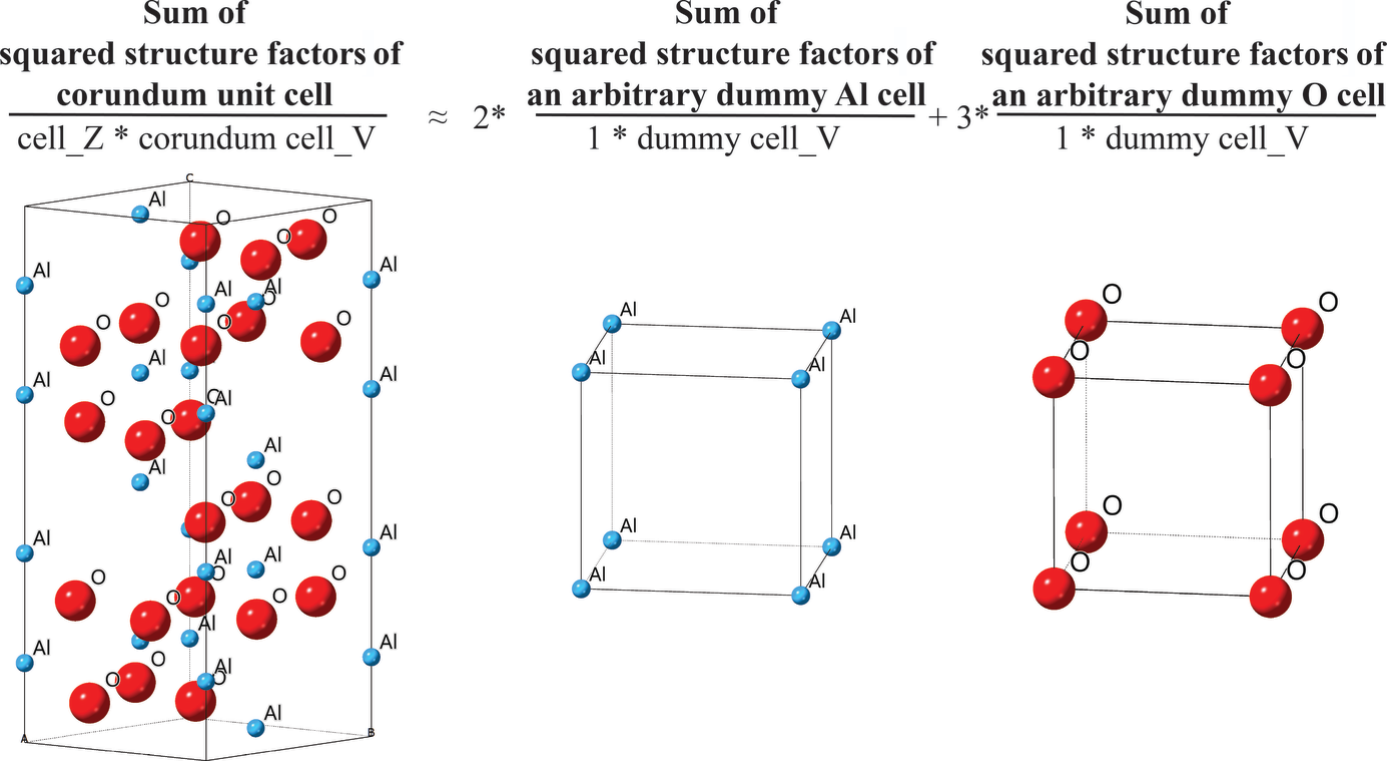
Example of the MSP method applied on corundum (α-Al_2_O_3_). The sum of squared structure factors of an Al_2_O_3_ molecule (unit-cell scattering power divided by the cell volume ‘cell_V’ and the number of molecules in the unit cell ‘cell_Z’) can be estimated by the sum of squared structure factors of two imaginary Al crystals and three imaginary O crystals in any arbitrary dummy cell, divided by the dummy cell volume (atomic scattering power).

This means that the molecular scattering power of phase *k* [the middle part of equation (16), hereafter denoted as ‘mol_f2_*k*_’], which is the unit-cell scattering power divided by the cell volume *V_k_* and the number of molecules in the unit cell *Z_k_*, can be approximated by the sum of squared structure factors of the *A_k_* arbitrary dummy cells divided by the dummy cell volume *V*_d_, as schematized in Fig. 2 ▸. Since the dummy cells contain only a single constituent atom each at their origins, the part in brackets of equation (16) is, in fact, the atomic scattering power of each constituent atom (sum of the squared product of the atomic form factor and its atomic displacement parameter).

Substituting equation (16) back into equation (3), we have
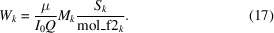


The weight percentage of phase *k* in a *K*-crystalline-phase mixture can, therefore, be expressed as
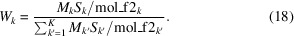


With 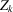 and 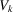 cancelled out, the expression of equation (18) is much simpler than the form proposed by Li *et al.* (2022 ▸) [equation (18) therein], meaning that it is not necessary to know the number of molecules in the unit cell *Z_k_*, the lattice parameters or the unit-cell volume *V_k_* when applying the MSP method to perform QPA for an unknown crystalline phase. Comparing this MSP method [equation (18)] with the conventional Rietveld QPA [equation (5)] reveals their relationship:
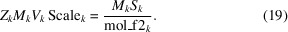


Equation (19) in fact conveys a similar concept to the intensity–composition equation of DDM (Toraya, 2021 ▸): the weight of a phase equals its diffraction intensity divided by its scattering power per unit mass. By comparing equation (19) with equation (10), we can derive the relationship between the conventional Rietveld QPA, the *C_k_*-corrected DDM and the MSP method:
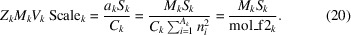


It is easy to see from equation (20) that the molecular scattering power mol_f2_*k*_ is equivalent to the ‘*C_k_*-corrected sum of squared electron numbers’ in the *C_k_*-corrected DDM approach. As pointed out in the explanation of equation (16), mol_f2_*k*_ is essentially a sum of atomic scattering powers (sum of the squared product of atomic form factor and atomic displacement parameter), which change with radiation wavelength for a fixed 2θ range, while the sum of 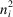 value used in DDM is merely a constant. Therefore, it is necessary to apply the proposed *C_k_* correction for DDM in order to reduce the discrepancies between the sum of squared electron numbers and mol_f2_*k*_. Equation (19) allows the incorporation of the MSP method for phase *k* of unknown crystal structure into the conventional Rietveld QPA routine.

### 2.4. Calculation of molecular scattering power mol_f2

**Figure 3:**
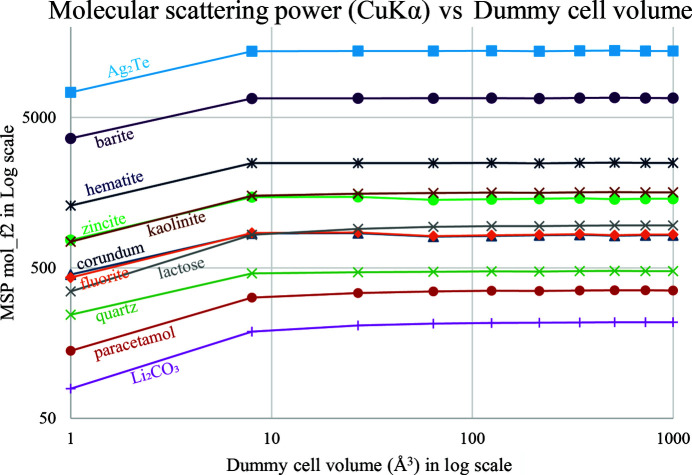
The molecular scattering powers of several crystalline phases under Cu *K*α radiation plotted against the volumes of dummy cubic cells used to calculate them.

The molecular scattering power mol_f2 of any chemical formula can be conveniently calculated through the right-hand term of equation (16) in the *TOPAS* software. Using the INP template provided in Section S1 of the supporting information, it took a laptop (Intel i7-1185 G7 @ 3 GHz 1.8 GHz, 16 GB RAM) less than 1 s to calculate the scattering power of the Al_2_O_3_ mol­ecule and save it into a mol_f2_corundum.inc file for the subsequent QPA INP to call. The MSP mol_f2 values for several crystalline phases are plotted against the used dummy cell volumes *V*_d_ in Fig. 3 ▸.

It is easy to see from Fig. 3 ▸ that, except for the smallest dummy cell of 1 Å^3^ (not larger than the Al or O atomic volumes), the MSP mol_f2 values are almost constant no matter what sizes of dummy cell were used to calculate them. This validates equation (14). Some fluctuations of zincite, corundum and fluorite are believed to be due to the ‘termination effect’ in calculating the sum of structure factors (Toraya, 2022 ▸). The MSP mol_f2 values are stabilized when a large dummy cell (*e.g.* a cubic cell of lattice parameter *a* = 10 Å) is used to generate many *hkl* reflections.

## 3. Implementations in the *TOPAS* software

It has been proposed that the *TOPAS* keyword numerical_area could be used to implement DDM. However, numerical_area was not designed to remove the LP factor (see Appendix *A*[App appa]). Therefore, it is not equivalent to the *S_k_* parameter in DDM calculation (Toraya, 2017 ▸). The following analysis steps are proposed to implement the *C_k_*-corrected DDM calculation and the MSP method in *TOPAS* v7 using the I parameters in Pawley phase hkl_Is and peaks phase xo_Is fitting.

### 3.1. Analysis steps for *C_k_*-corrected DDM

(*a*) A *TOPAS* template cal_C.inp exporting the *C_k_* value for a crystalline phase is described in Section S2 of the supporting information. Using this template, readers can calculate the *C_k_* value of any analyte for the scanned wavelength and 2θ range. The result is saved into a .inc file for the subsequent QPA INP to call.

(*b*) Apply Pawley or Le Bail fitting using the hkl_Is model to extract the peak area of the crystalline phase of partially known structure, together with Rietveld fitting for other phases of known crystal structure. The sum of all the extracted and fixed I values in the hkl_Is phase is assigned to a parameter *S_k_*. The Scale keyword is not used in hkl_Is phases and hence equals 1.

(*c*) Calculate the molecular weight *M_k_* and the total squared electron numbers for each atom in the molecule 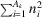. With the *C_k_* value determined in step (*a*), a DDM_aS_on_C value for this phase is calculated as 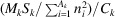, which is equivalent to the *Z_k_M_k_V_k_* Scale_*k*_ factor in Rietveld QPA. Use the value of DDM_aS_on_C / cell_volume as the cell_mass (*Z_k_M_k_*) in the hkl_Is model.

(*d*) Execute the same .inp file again; the Rietveld QPA routine in *TOPAS* will report the weight percentages of all the component phases, including the DDM-modelled unknown phase, according to equation (5).

### 3.2. Analysis steps for molecular scattering power method

(*a*) As shown in Section 2.4, use the *TOPAS* INP template described in Section S1 of the supporting information to calculate the MSP mol_f2 value for the chemical formula (molecule) of any phase of partially or no known crystal structure, providing its chemical formula is known.

(*b*) Same as Step (*b*) in Section 3.1, if the lattice parameters are known for the target phase. Otherwise use the xo_Is model to fit the peak area of the phase of no known structure, together with Rietveld fits for other phases of known crystal structure. The sum of all the extracted and fixed I values in the xo_Is model is assigned to a parameter 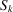. The Scale keyword is not used in the xo_Is phase and hence equals 1.

(*c*) Calculate the molecular weight *M_k_*. With the MSP mol_f2 value determined in step (*a*), the value of 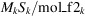 can be used as the cell_mass of the xo_Is model, in which its cell volume is set to 1, according to equation (19). In the case of using the hkl_Is model, 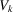 is calculated from the known lattice parameters. Therefore, use the value of (*M_k_S_k_*/mol_f2_*k*_)/cell_volume as the cell_mass of the hkl_Is model.

(*d*) Same as Step (*d*) in Section 3.1.

## 4. Examples

### 4.1. Test on the calculated XRD pattern for a 1:1 weight mixture of Ag_2_Te and Li_2_CO_3_

This extreme counter-example was used in He & Li’s (2022 ▸) comment on DDM, which highlighted the fact that *C* values are phase dependent. Ignoring their differences may lead to wrong QPA results. The current analysis steps propose to include *C_k_* into the DDM calculation to make it compatible with the conventional Rietveld QPA routine.

**Figure 4:**
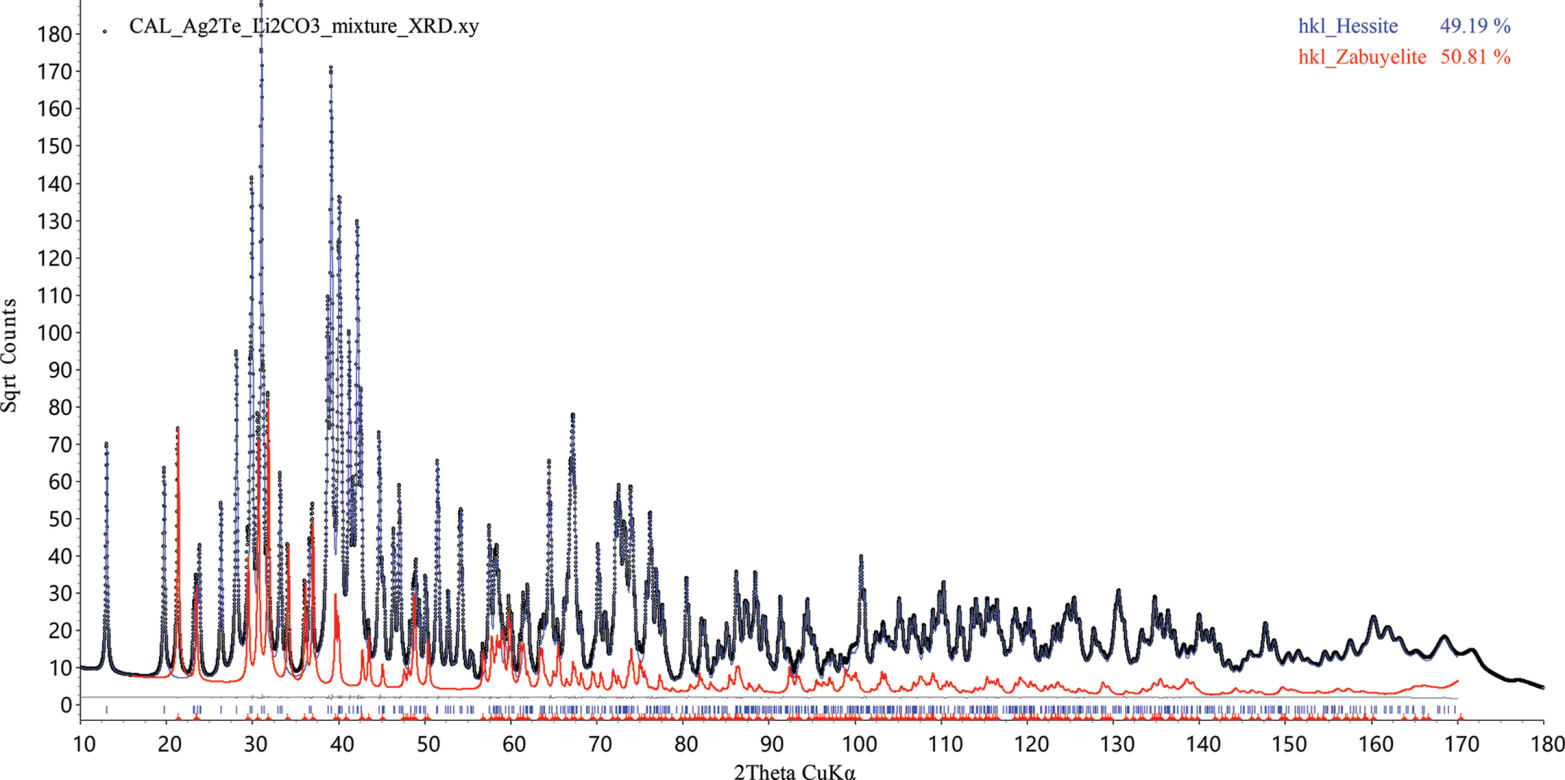
Full pattern refinement of the calculated XRD pattern of 1:1 weight ratio of hessite (Ag_2_Te) and zabuyelite (Li_2_CO_3_), using the *C_k_*-corrected DDM for both phases (D D in Table 1 ▸). The calculated data are shown as black dots. The contribution of hessite is shown as the blue curve, while that of zabuyelite is highlighted as the red curve.

The *TOPAS* file Ag2Te_Li2CO3_mixture.inp and the calculated XRD pattern for a 1:1 weight mixture of Ag_2_Te and Li_2_CO_3_ are available in the supporting information. The calculated *C_k_* value for hessite, Ag_2_Te, is 1.91, while the *C_k_* value for zabuyelite, Li_2_CO_3_, is 0.79. The INP file contains str models and hkl_Is models for both phases. In total, four combinations of model choices are considered, and the QPA results reported in the corresponding OUT file are summarized in the first four columns of Table 1 ▸. Since the weight percentage sum of Ag_2_Te and Li_2_CO_3_ phases is 100%, only Ag_2_Te wt% results are shown in Table 1 ▸. The full-pattern fits using the *C_k_*-corrected DDM for both phases are shown in Fig. 4 ▸.

Table 1 ▸ indicates that, by bringing back the *C_k_* correction into equation (9), accurate or reasonable QPA results are achieved even for this extreme counter-example, in contrast to the wrong QPA results (more than 20 wt% discrepancy) obtained through the original DDM as pointed out by He & Li (2022 ▸). This example serves the purpose of validating the effectiveness of equation (9), *i.e.* the rectification effect of *C_k_* on top of the original DDM.

Analogously, the MSP method proposed in this paper is tested on this example, and the QPA results are reported in the last three columns of Table 1 ▸. The MPS method is also able to deliver QPA results of reasonable accuracy for this example, validating the effectiveness of equation (18). The full-pattern fitting modelled by the same hkl_Is phases is identical to that in Fig. 4 ▸ and is hence omitted. The implemented MSP QPA equations are also stored in the same *TOPAS* INP file in the supporting information. A global level selection is devised to allow user toggling between the *C_k_*-corrected DDM and the MSP mol_f2 method.

### 4.2. Test on the IUCr round robin CPD-1 series dataset

Eight publicly available XRD patterns (from CPD-1A.raw to CPD-1H.raw, see *Data availability* section) measured for three-phase mixtures (corundum, fluorite and zincite) of known weighed mass percentages (Madsen *et al.*, 2001 ▸) were used to test the accuracy of the analysis steps proposed in Section 3. The file header of the *TOPAS* INP (Cpd-1a.inp to Cpd-1h.inp in the supporting information) allows readers to toggle between the *C_k_*-corrected DDM and the MSP method. The *S_k_* values were set using *TOPAS* keyword prm_with_error to propagate intensity errors into those of the QPA results. Each phase has both structure str and hkl_Is models set up for selection. The calculated *C_k_* values for corundum, fluorite and zincite are 1.48, 1.39 and 1.49, respectively. The calculated MSP mol_f2_k_ values for them are 825.609, 832.917 and 1509.435, respectively. The same thermal vibration parameters beq used in the Rietveld QPA were used in the calculation of these values. The macros AW and AN return atomic weight and atomic number, respectively (available in supporting information Section S3).

The QPA results using all eight combinations of Rietveld QPA and the *C_k_*-corrected DDM for these three-phase mixtures are summarized in the ‘*C_k_*-corrected DDM’ columns of Table 2 ▸, together with the weighed percentages and the QPA results from the original DDM method without *C_k_* corrections. These QPA results are also plotted on a ternary phase diagram (Fig. S1) to visualize their accuracies and precisions.

Table 2 ▸ and Fig. S1 show that the QPA results from the proposed *C_k_*-corrected DDM hybrid with Rietveld QPA are generally more accurate than the results from the original DDM without *C_k_* correction. This set of QPA results suggest that *C_k_* correction for each phase improves DDM accuracy. However, if individual *C_k_* values (requiring crystal structure) are not available, omitting this correction does not change the QPA results too much (<3 wt%) for this dataset, in which the average atomic numbers between phases are not as far apart as they are in the first example in Section 4.1.

Analogously, the QPA results using all eight combinations of Rietveld QPA and the MSP method using Pawley fitting (hkl_Is) for these three-phase mixtures are summarized in the ‘MSP using hkl_Is’ columns of Table 2 ▸. These QPA results are also plotted on a ternary phase diagram (Fig. S2) to visualize their accuracies and precisions. It can be seen from Table 2 ▸ and Fig. S2 that, compared with *C_k_*-corrected DDM, although slightly worse QPA accuracies and precisions are observed, their deviations from the weighed weight percentages are scarcely higher than 3 wt%. The benefit of this MSP method is that it totally eliminates the requirement of knowing the crystal structure (atomic positions).

### 4.3. Applying the MSP method for phases of unknown lattice parameters

**Figure 5:**
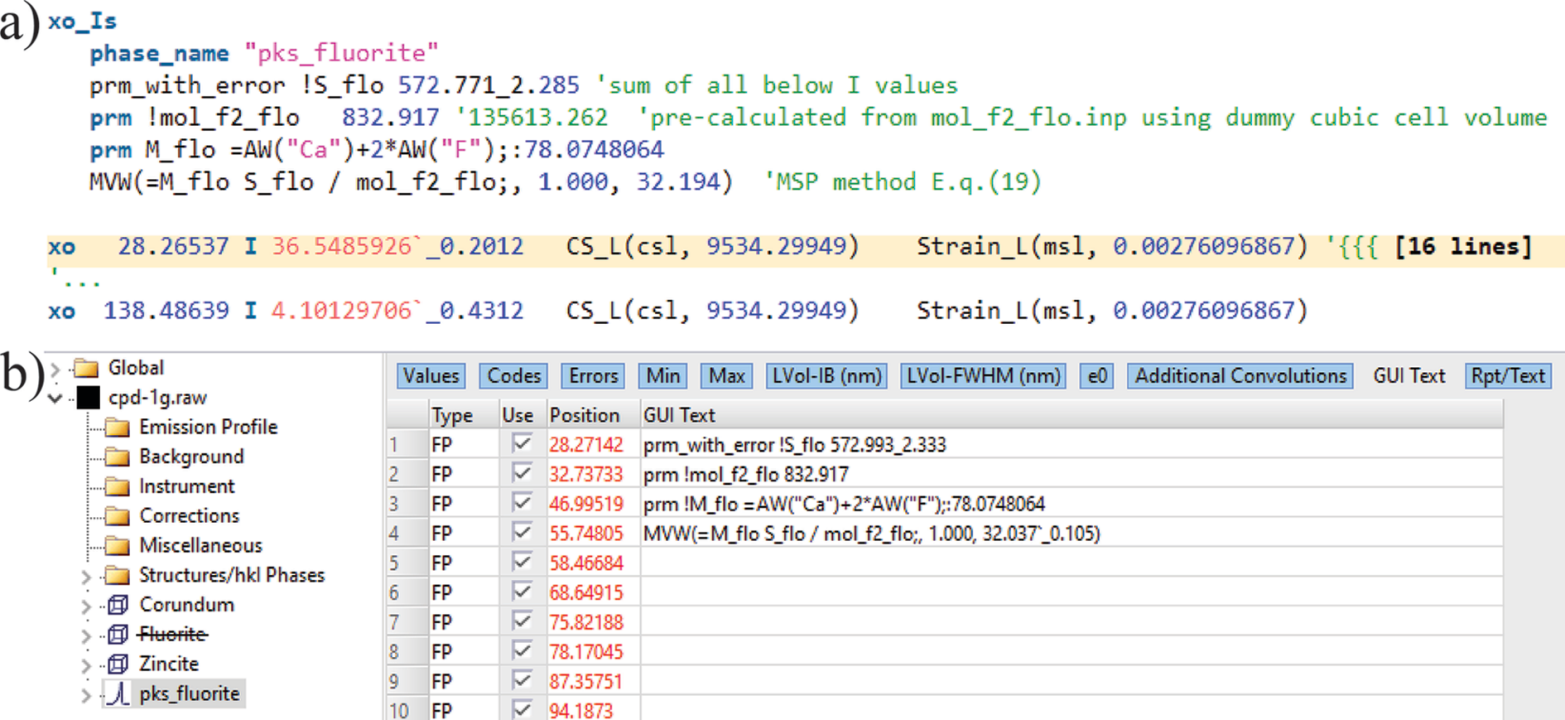
Equation (19) of the MSP method implemented in *TOPAS* (*a*) Launch Mode and *b*) GUI Mode, to quantify a phase of unknown lattice parameters.

The procedure of Section 3.2 is also tested on the CPD-1 series dataset, assuming the lattice parameters of the target phases are also not known. The *TOPAS* INP files (Cpd-1a_xo_Is.inp to Cpd-1h_xo_Is.inp) in the supporting information include equation (19) derived for the target phases, without using any lattice parameter, as shown in Fig. 5 ▸(*a*). The same operation in *TOPAS* GUI Mode is shown in Fig. 5 ▸(*b*). The peaks phase model xo_Is [also called the Type A fitting function by Toraya (2018 ▸, 2019 ▸)] is used in these examples.

The peak positions in the xo_Is model are not constrained by lattice parameters as they are in Pawley fitting (hkl_Is model). Owing to the inevitable peak overlapping of multiphase powder patterns, simultaneous fitting of two or more unknown phases using peaks phases (xo_Is model) will not partition the intensity correctly. Therefore, only four combinations of Rietveld QPA and the MSP method using the xo_Is model for the three-phase mixtures in the CPD-1 series were tested, and their results are summarized in the ‘MSP using xo_Is’ columns in Table 2 ▸. These QPA results are also plotted on a ternary phase diagram in Fig. S3. From Table 2 ▸ and Fig. S3, compared with the MSP method using Pawley fitting (hkl_Is), similar levels of QPA discrepancy from weighed percentages (<3 wt%) are observed.

Figs. S1–S3 show that all methods, including the conventional Rietveld method, overestimate corundum. This is known to be due to the micro-absorption effect, in which the high-mass-absorption phase tends to be underestimated, while the low-mass-absorption phase tends to be overestimated.

### 4.4. Applying the MSP method for a phase of no known lattice parameter or chemical composition

Toraya (2017 ▸) pointed out [equation (20) therein] that it is still possible to quantify a phase of unknown crystal structure and unknown chemical composition if all the other phases have known scattering power per unit weight 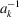. This was based on the idea of treating the whole mixture as a single phase and deriving its chemical composition from either (1) the starting raw materials before reactions, (2) other elemental analysis techniques, *e.g.*X-ray fluorescence spectroscopy (XRF) *etc.*, or (3) scenarios elaborated in Section 3 of Toraya (2017 ▸).

Since equation (20) of the present paper has shown that the term *M_k_*/mol_f2_*k*_ in the current MSP method is essentially the same as *a_k_*/*C_k_* in DDM, a similar approach can be proposed and tested (see Table 3 ▸). The chemical composition of sample ‘cpd-1h’ based on the publicly available XRF data (see the *Data availability* section) is shown in the first row (left) of Table 3 ▸. Using the *TOPAS* INP template of supporting information Section S1, it is easy to calculate its ratio of *M_k_*/mol_f2. With the sum of LP-corrected total intensity *S_k_* of the ‘cpd-1h’ pattern, the value of *M_k_S_k_*/mol_f2 for the whole mixture sample can be calculated (second last row of Table 3 ▸), which is close to the sum of the same parameter calculated for the three individual phases on the right side of Table 3 ▸. Therefore, if any individual phase has unknown chemical composition (unknown *M_k_* or mol_f2), it is still possible to derive its *M_k_S_k_*/mol_f2 value by subtracting the *M_k_S_k_*/mol_f2 value of other known phases from the total *M_k_S_k_*/mol_f2 value of the whole mixture sample. The weight percentages are just normalized *M_k_S_k_*/mol_f2 values.

### 4.5. Disordered kaolinite

Kaolinite KGa-2 is a poorly crystalline kaolinite source clay with stacking disorder (Sakharov *et al.*, 2016 ▸). The diffraction pattern of the mixture of this standard and 20 wt% corundum after homogenizing in a McCrone micronizer was measured using a Bruker D8 Advance diffractometer under Co *K*α radiation (40 kV, 40 mA). Dynamic Beam Optimization optics, including a variable divergence slit illuminating a 10 mm sample length and an automatic air-scattering knife above the sample, were used to eliminate air-scattering background and sample holder background, which helps in direct extraction of the scattering intensity of poorly crystalline phases. Soller slits (2.5°) were used on both primary and secondary sides of the beam path. A LynxEye XE-T detector (1D mode) was used to collect the diffraction signal from the sample spun at 15 rpm around the sample surface normal axis (to improve statistics) from 2 to 90° 2θ at a 0.015° step size in 1 h.

**Figure 6:**
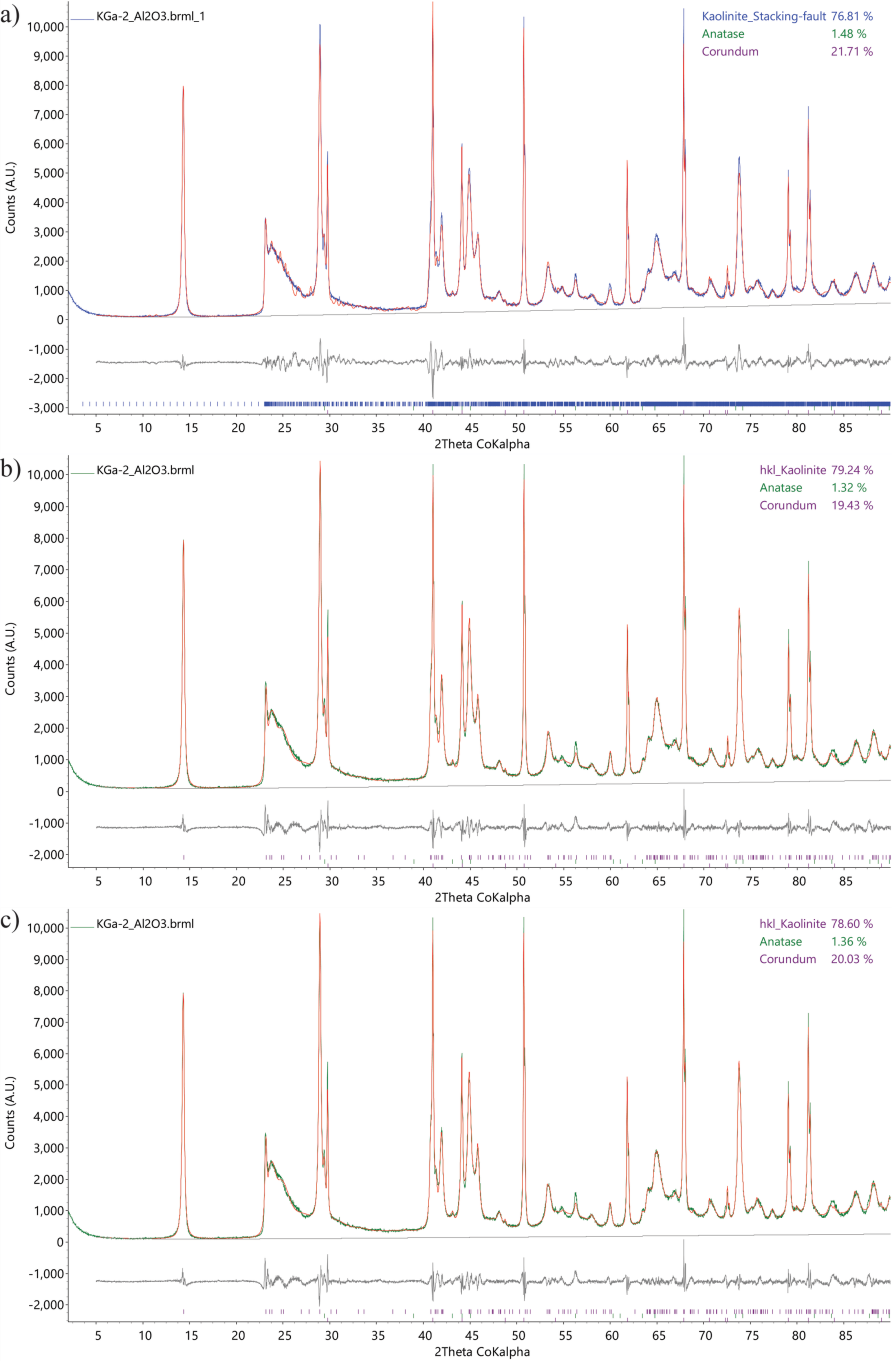
(*a*) Using the str structure model for kaolinite together with spherical harmonics for preferred orientation corrections, stacking fault modelling, crystallite size broadening and micro-strain broadening. *R*_wp_ 8.03%, goodness of fit (GOF) 2.67. (*b*) Using hkl_Is Pawley fitting and the proposed *C_k_*-corrected DDM calculation [Fig. 7(*b*)] to quantify the kaolinite KGa-2 in synthetic mixture. *R*_wp_ 6.63%, GOF 2.19. (*c*) Same fitting except MSP QPA equations [Fig. 8(*a*)] are used. All fits used first-order Chebyshev background.

Fig. 6 ▸ compares the whole-pattern fits from Rietveld QPA using the kaolinite str model, from the *C_k_*-corrected DDM calculation and from the MSP method (hkl_Is), for the above-described synthesized mixture of kaolinite KGa-2 source clay spiked with 20 wt% corundum. The QPA results obtained from each method are shown in the top right corners. Fig. 6 ▸(*a*) shows Rietveld QPA using the str structure model (Lee & Xu, 2020 ▸) with various peak profile corrections, including spherical harmonics correction for preferred orientation (Järvinen, 1993 ▸; Bruker, 2024 ▸), stacking fault modelling^2^ (Ufer *et al.*, 2004 ▸; Wang *et al.*, 2012 ▸; Bruker, 2014 ▸; Coelho *et al.*, 2016 ▸; Bruker, 2017 ▸), and crystallite size broadening and micro-strain broadening in *TOPAS* Launch Mode (INP file available in the supporting information). Figs. 6 ▸(*b*) and 6 ▸(*c*) show similar fits using the same hkl_Is model in GUI Mode, which generate the same *S_k_* value (sum of LP-factor-corrected intensities). Stephens’ anisotropic peak broadening model (Stephens, 1999 ▸) for triclinic space groups was used in the ‘Microstructure’ tab of GUI Mode to fit the kaolinite asymmetric non-basal reflections. The difference between Figs. 6 ▸(*b*) and 6 ▸(*c*) is only in their QPA equations as described below, hence the slightly different phase weight percentages reported in their top-right corners. The *TOPAS* PRO files implemented with the *C_k_*-corrected DDM and the MSP method (hkl_Is) are also available in the supporting information.

**Figure 7:**
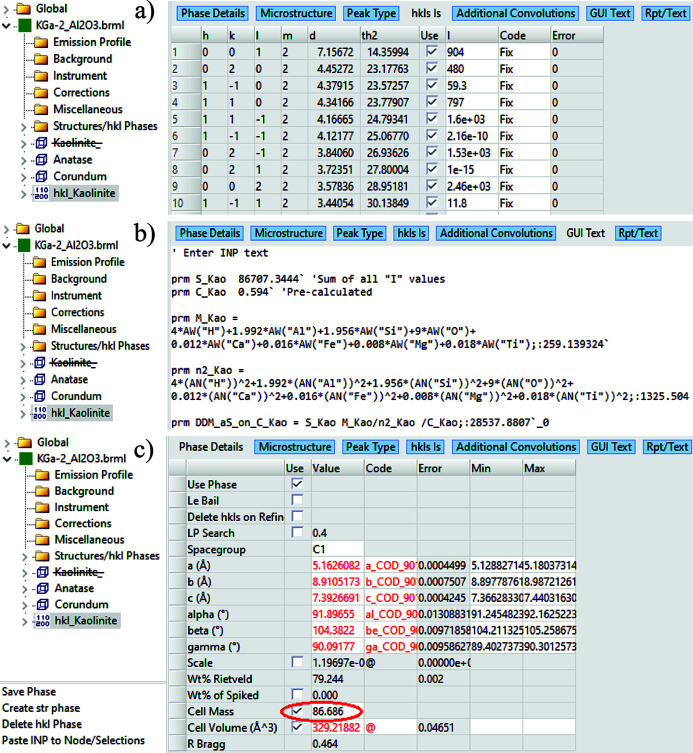
*C_k_*-corrected DDM calculation in *TOPAS* GUI Mode: step (*a*) sum all I values from Pawley or Le Bail fitting using an hkl_Is phase; step (*b*) assign the sum to the parameter S_Kao; derive the DDM_aS_on_C value from the pre-calculated correction factor C_Kao, the molecule weight M_Kao and the sum of electron number squared for all atoms in the molecule n2_Kao; step (*c*) type in the value of DDM_aS_on_C/Cell Volume into the ‘Cell Mass’ box for this hkl_Is phase. Run the refinement again to obtain QPA results for all phases in this mixture.

Since *TOPAS* v7, a ‘GUI Text’ tab has been added to its GUI, allowing users to implement custom equations within the *TOPAS* GUI Mode. The implementations of the *C_k_*-corrected DDM method [equation (10)] and the MSP method [equation (19)] in the *TOPAS* GUI Mode for this example are shown in Figs. 7 ▸(*b*) and 8 ▸(*a*), respectively.

To minimize peak-area correlation with the background, only the first-order Chebyshev polynomials were refined for background across all methods. Both new approaches yielded comparable QPA results to those from the conventional Rietveld QPA, which is significantly more complex in disordered structural modelling and corrections. Note that the MSP method was design for analysing full-range XRD patterns (Li *et al.*, 2022 ▸; Li & He, 2023 ▸). Applying the method to a relatively narrow 2θ range (2–90°) in this example may reduce its accuracy. Nevertheless, the discrepancies between the quantified corundum weight percentages and its known values are lower than 2 wt%.

**Figure 8:**
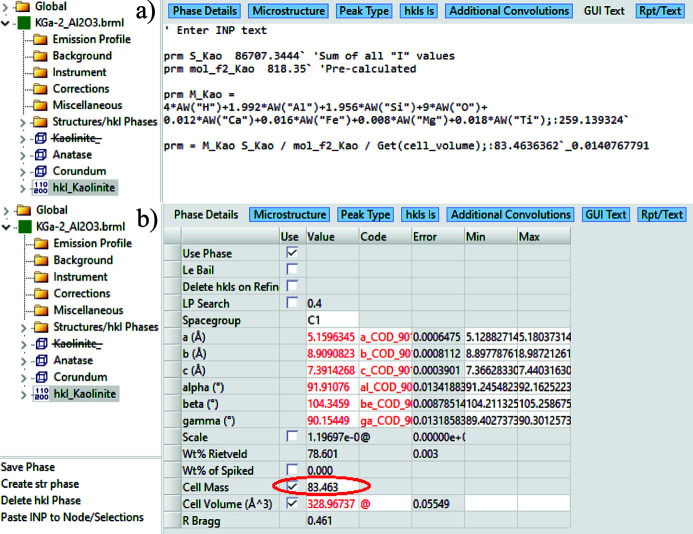
MSP method in *TOPAS* GUI Mode: (*a*) assign the sum of I from hkl_Is fitting to the parameter S_Kao; calculate the M_Kao S_Kao / mol_f2_Kao / Get(cell_volume) value from the molecule weight M_Kao and the pre-calculated molecular scattering power mol_f2_Kao; (*b*) input the above-calculated value of M_Kao S_Kao / mol_f2_Kao / Get(cell_volume) into the ‘Cell Mass’ box for this hkl_Is phase. Run the refinement again to obtain QPA results for all phases in this mixture.

In the example shown in Fig. 8 ▸, the unit-cell volume is effectively cancelled out when the *TOPAS* QPA routine calculates the product of cell mass and cell volume. As a result, it is not explicitly required in the MSP QPA equation, *i.e.* equation (19). In this case, the unit-cell parameter is used solely to achieve a good fit to the kaolinite KGa-2 pattern, ensuring accurate *S_k_* extraction. The next example will demonstrate the scenario in which the lattice parameters of the unknown crystal structure phase are not used through the MSP method (xo_Is).

### 4.6. Disordered chlorite

The aforementioned corrections applied to the crystal structure in conventional Rietveld QPA may not adequately account for other complex structural disorders. An illustrative example is the clay mineral ripidolite, which represents an intermediate chlorite group member between chamosite (Fe-rich) and clinochlore (Mg-rich). Chlorite CCa-2 is a crystalline ripidolite source clay with cationic disorder (Gailhanou *et al.*, 2009 ▸). The chemical formula of CCa-2 derived from the chemical composition reported therein is Ca_0.022_(Fe_3.682_Mg_5.650_Mn_0.022_K_0.013_Na_0.039_Ti_0.117_P_0.003_)(Si_5.040_Al_4.753_)O_20_(OH)_16_, with a molecular weight of 1214.033 g mol^−1^. The MSP of this formula is calculated as 4303.128, using the *TOPAS* file cal_mol_f2_CCa-2_Co.inp (available in the sup­port­ing information).

The diffraction pattern of the mixture of this standard and 20 wt% corundum after homogenizing in a McCrone micronizer was measured using a Bruker D8 Endeavor ECO diffractometer under Co *K*α radiation (35 kV, 28 mA). Dynamic Beam Optimization optics, including a variable divergence slit at 15 mm sample illumination length and an automatic air-scattering knife above the sample, were used to eliminate air-scattering background and sample holder background, which helps in direct extraction of the scattering intensity of poorly crystalline phases. Soller slits (4.1°) were used on both primary and secondary sides of the beam path. A LynxEye XE-T detector (1D mode) was used to collect the diffraction signal from the sample spun at 15 rpm around the sample surface normal axis (to improve statistics) from 2 to 90° 2θ at a 0.015° step size in 1 h.

**Figure 9:**
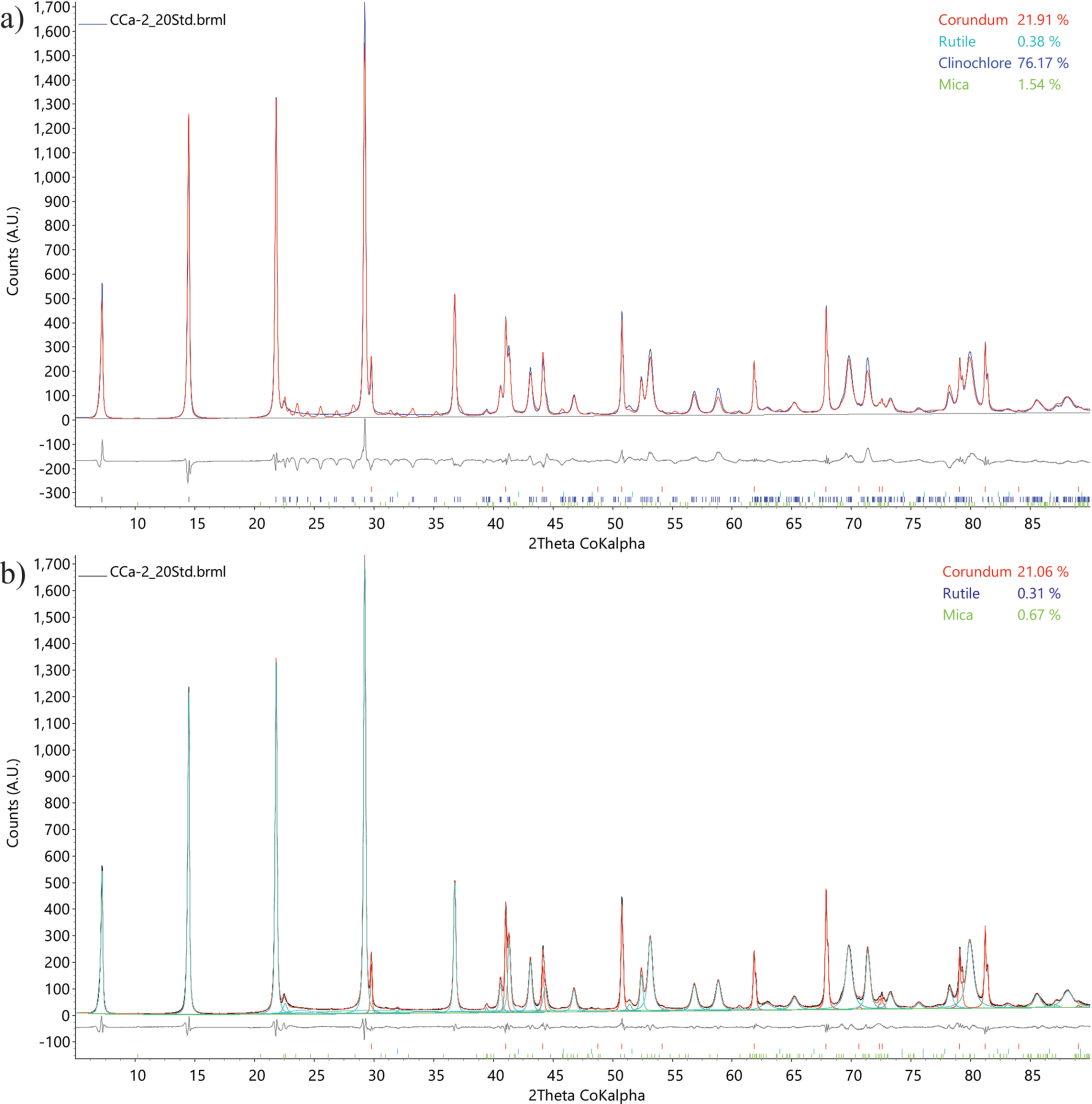
(*a*) Using the str structure model for ripidolite CCa-2 together with Stephens’ anisotropic peak broadening model. *R*_wp_ 13.76%, GOF 12.21; (*b*) Using xo_Is peak fitting for the MSP method (Fig. 10 ▸). *R*_wp_ 5.45%, GOF 4.87. All fits used a first-order Chebyshev background.

Fig. 9 ▸ compares the whole-pattern fitting from Rietveld QPA using the clinochlore str model (Zanazzi *et al.*, 2007 ▸) and from the MSP method (xo_Is) for the above-described synthesized mixture of ripidolite CCa-2 source clay spiked with 20 wt% corundum. As can be seen from Fig. 9 ▸(*a*), many non-basal reflections calculated from the clinochlore str crystal structure that ought to be present between 20 and 30° 2θ are completely missing in the measured data. Stephens’ anisotropic peak broadening correction is applied but still not able to handle such a large discrepancy. In contrast, a much superior fit is achieved in Fig. 9 ▸(*b*) using the MSP method (xo_Is), because it allows more flexible individual peak profiles to be refined.

**Figure 10:**
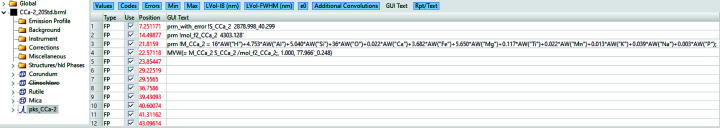
In the last macro, MVW, the first parameter is ‘Cell mass’, written as *M_k_S_k_*/mol_f2_*k*_; the 2nd parameter 1.000 represents the unit-cell volume of the xo_Is model; the third parameter shows the refined QPA result for ripidolite CCa-2: 78.0 (2) wt%.

The QPA results in the top-right corner of Fig. 9 ▸(*a*) overestimate corundum. In contrast, Fig. 9 ▸(*b*) shows slightly more accurate QPA results for corundum obtained through the model-free MSP (xo_Is) approach. The calculated weight percent of ripidolite CCa-2 [78.0 (2) wt%] is shown in the MVW macro in Fig. 10 ▸.

In Fig. 10 ▸, the sum of I values extracted using the xo_Is model pks_CCa-2 is assigned to *S_k_* using the prm_with_error keyword to take into account the errors from the intensity extraction. With the molecular scattering power mol_f2_*k*_ calculated above and the formula weight *M_k_* calculated using AW macros, *M_k_S_k_*/mol_f2_*k*_ [equation (19)] can be used as the cell_mass in the MVW macro, as described in Section 3.2 step (*c*).

The CCa-2_20Std.pro file used for this calculation is provided in the supporting information.^3^ In this example, the proposed MSP method employs the peaks phase xo_Is model to extract the diffraction intensities of CCa-2 and calculate its molecular scattering power only from its published chemical composition. No information on lattice parameters or atomic positions was used. The MSP method is both simpler and more accurate than conventional Rietveld QPA for quantifying the poorly crystalline clay mineral CCa-2 ripidolite.

## 5. Discussion

In the diffraction pattern of a multi-phase mixture, peak overlaps are inevitable. By using crystal structural models for known crystalline phases, we can subtract their intensities from the overlapping peaks, allowing the remaining intensity to be allocated to a Pawley or Le Bail fit for a poorly crystalline or disordered phase. This combined refinement was also adopted by Toraya (2018 ▸, 2019 ▸), who used peaks phase xo_Is and Pawley phase hkl_Is (Type A fitting function therein) and Rietveld modelling or intensity data from databases (Type B fitting function therein) alongside pre-measured scans using the *FULLPAT* method (Chipera & Bish, 2002 ▸) (Type C fitting function therein), all integrated within a single refinement. This approach is considered superior to applying Type A fitting functions to all phases, where the partitioning of overlapping peak intensities among contributing phases heavily depends on the partitioning strategy. The iterative ‘volume-proportional partitioning’ strategy (Toraya, 2016 ▸) still requires an initial assumption of equal partitioning, which may face problems when the primary diffraction peaks of an unknown phase significantly overlap with those of other phases.

In conventional Rietveld QPA, the structure factor of each *hkl* reflection can be calculated from the known crystal structures of the constituent phases, allowing for the reconstruction of XRD patterns (Hill & Howard, 1987 ▸). In addition to determining the weight percentages of component phases (QPA), Rietveld QPA also provides valuable information on crystallite size, microstrain, preferred orientation, atomic displacement parameters, atomic occupancies and atomic positions, among other structural characteristics. In this context, knowledge of the crystal structure serves as a sufficient but not a necessary condition for QPA. QPA can still be performed as long as the total structure factors, or the scattering power per unit mass of the analyte, can be either calculated or experimentally calibrated.

The application field of the currently proposed *C_k_*-corrected DDM is limited to poorly crystalline phases, as it still relies on the crystal structure being close enough to disorder free to calculate the *C_k_* values. In contrast, the MSP method totally eliminates the need for information on atomic positions, lattice parameters, unit-cell volume or the number of mol­ecules per unit cell. Instead, it requires only the chemical composition of the unknown phase.

Both of the proposed methods are classified as ‘direct’ QPA methods (Madsen *et al.*, 2011 ▸), which require accurate separation of pattern background from phase contributions. In the present examples, the proposed methods are shown to be effective for analysing XRD powder patterns collected using a variable divergence slit. The use of Dynamic Beam Optimization optics suppresses non-sample scattering background, thereby facilitating the direct extraction of the scattering intensity of poorly crystalline phases. Additionally, a very low order Chebyshev polynomial (only first order) was used to model the pattern background, further reducing the correlation of background with intensity *S_k_* extracted via Pawley fitting or peaks phase fitting. In the case of applying a ‘direct’ QPA method to an amorphous phase, the separation of phase contributions from background becomes more challenging and typically necessitates experimental calibration. In such cases, the PONKCS method and the internal standard method remain more efficient alternatives. Effective background determination methods for multi-phase mixtures have been reported (Madsen *et al.*, 2011 ▸; Toraya, 2019 ▸; Toraya & Omote, 2019 ▸) and can be adopted.

The proposed *C_k_*-corrected DDM and MSP methods both omit the electron density in interatomic bonds, which may explain their discrepancy with the Rietveld method. Rietveld QPA is still considered to be the most accurate QPA method, supported by Table 2 ▸, in which methods with more ‘R’ yield more accurate QPA results. However, if the accuracies of QPA results obtained from the two currently proposed *C_k_*-corrected DDM and MSP methods (absolute deviation within ±3 wt%) are deemed acceptable, they could be widely applied to quantify poorly crystalline, disordered phases or partially or no known crystal structures. Such cases would otherwise require complex explicit structural modelling or corrections, or laborious experiment calibration. The two proposed methods thus hold significant potential for, especially, industrial applications.

## 6. Conclusion

Analysing the equations of the direct derivative method (Toraya, 2016 ▸) reveals that the *a_k_S_k_*/*C_k_* term in DDM terminology is essentially equivalent to the *Z_k_M_k_V_k_*Scale_*k*_ factor in the conventional Rietveld QPA method. Incorporating individual *C_k_* factors for different phases enhances the accuracy of the original DDM calculations, particularly for mixtures with high atomic-number contrast. Similarly, analysing the equations of the original unit-cell scattering power method (Li *et al.*, 2022 ▸) enables its simplification into a molecular scattering power method, where *M_k_S_k_*/mol_f2_*k*_ is equivalent to the *Z_k_M_k_V_k_*Scale_*k*_ factor in conventional Rietveld QPA. The MSP method eliminates the need for knowledge of the number of molecules per unit cell, lattice parameters and unit-cell volume. While the MSP method endorses the concept of *C_k_*-corrected DDM, the MSP method does not require *C_k_* calculations to relate to the squared electron numbers of atoms in the molecule. Instead, the MSP method determines molecular scattering power by summing the atomic scattering powers (the sum of squared product of atomic form factor and atomic displacement parameter).

## Supplementary Material

TOPAS. inp files and TOPAS macros developed for the proposed QPA methods. DOI: 10.1107/S1600576725004054/gue5001sup1.pdf

TOPAS implementations of both proposed methods used in all examples in the paper. DOI: 10.1107/S1600576725004054/gue5001sup2.zip

## Figures and Tables

**Figure 11:**
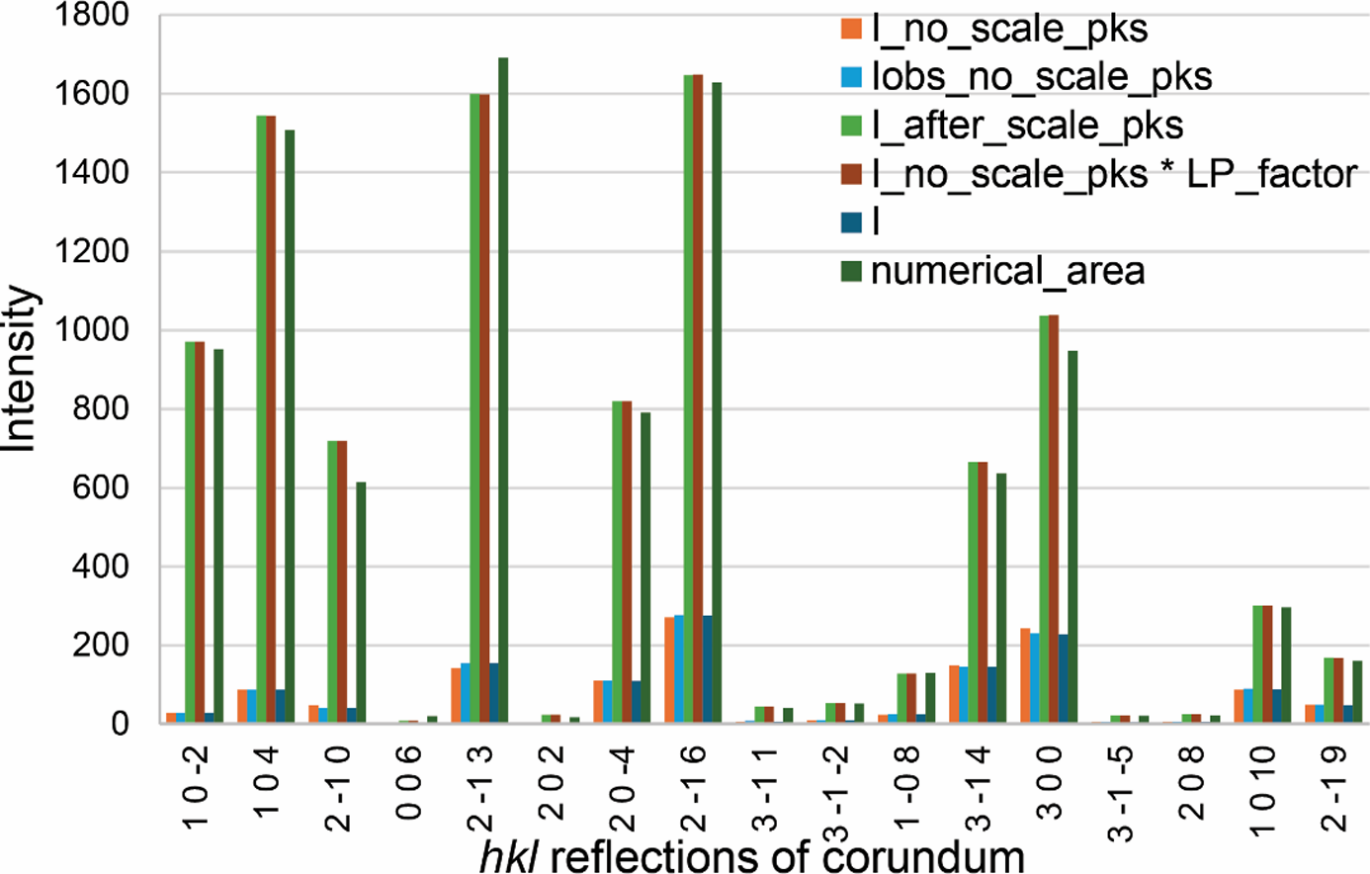
Comparison of the reserved intensity parameters in *TOPAS* from a refinement of the powder diffraction pattern corundum.raw in the IUCr CPD round robin. The five histograms for each *hkl* reflection follow the order of the legend.

**Table 1:** QPA results of the XRD pattern calculated from a 1:1 weight ratio of Ag_2_Te and Li_2_CO_3_ using different combinations of QPA methods: R – Rietveld QPA; D – *C_k_*-corrected DDM; f – MSP method

	Choices of QPA methods used for Ag_2_Te (hessite) and Li_2_CO_3_ (zabuyelite), respectively						
	R R	D R	R D	D D	R f	f R	f f
Ag_2_Te wt%	49.986 (7)	49.867 (5)	49.320 (1)	49.187 (1)	52.144 (1)	49.420 (5)	51.556 (1)

**Table:** R – Rietveld QPA; D – *C_k_*-corrected DDM; f – MSP method using Pawley fitting (hkl_Is); x – MSP method using peaks phases (xo_Is). Their orders represent the corresponding model used for corundum, fluorite and zincite, respectively. The column ‘D D D no *C_k_*’ stands for the original DDM calculation without *C_k_* correction. The numbers in brackets are the refinement errors aligned for the last decimal place.

			Rietveld	*C_k_*-corrected DDM							Original DDM
Sample ID	Weighed wt%		R R R	R R D	R D R	R D D	D R R	D R D	D D R	D D D	D D D no *C_k_*
CPD-1a	Corundum	1.15	0.09 (7)	0.11 (7)	0.10 (7)	0.12 (7)	0.57 (18)	0.57 (18)	0.59 (18)	0.59 (18)	0.70 (19)
	Fluorite	94.81	0.14 (8)	0.18 (10)	0.08 (8)	0.12 (10)	−0.34 (18)	−0.27 (18)	−0.41 (18)	−0.33 (19)	−0.7 (2)
	Zincite	4.04	−0.23 (4)	−0.29 (7)	−0.18 (4)	−0.24 (7)	−0.23 (4)	−0.30 (7)	−0.18 (4)	−0.26 (7)	−0.01 (8)
CPD-1b	Corundum	94.31	0.12 (4)	−0.11 (6)	0.32 (6)	0.11 (8)	0.14 (4)	0.05 (6)	0.35 (6)	0.18 (8)	0.41 (7)
	Fluorite	4.33	−0.14 (4)	−0.12 (4)	−0.35 (6)	−0.33 (6)	−0.16 (4)	−0.16 (4)	−0.38 (6)	−0.39 (6)	−0.62 (5)
	Zincite	1.36	0.02 (2)	0.23 (6)	0.03 (2)	0.23 (6)	0.02 (2)	0.20 (5)	0.03 (2)	0.21 (5)	0.22 (6)
CPD-1c	Corundum	5.04	0.75 (11)	0.67 (11)	0.80 (11)	0.64 (11)	2.5 (4)	2.5 (4)	2.4 (4)	2.4 (4)	2.4 (4)
	Fluorite	1.36	0.02 (4)	0.02 (4)	0.42 (10)	0.42 (10)	0.02 (4)	0.03 (4)	0.39 (10)	0.4 (1)	0.28 (9)
	Zincite	93.59	−0.76 (11)	−0.68 (11)	−1.21 (14)	−1.06 (14)	−2.5 (4)	−2.5 (4)	−2.8 (4)	−2.8 (4)	−2.7 (4)
CPD-1d	Corundum	13.53	1.03 (11)	1.09 (11)	1.17 (11)	1.23 (11)	0.9 (2)	1.0 (2)	1.1 (2)	1.1 (2)	1.6 (2)
	Fluorite	53.58	−0.59 (11)	−0.47 (11)	−1.02 (12)	−0.90 (12)	−0.54 (15)	−0.39 (15)	−0.98 (15)	−0.84 (16)	−2.51 (16)
	Zincite	32.89	−0.44 (9)	−0.62 (10)	−0.15 (9)	−0.32 (11)	−0.40 (11)	−0.59 (12)	−0.10 (11)	−0.28 (12)	0.92 (13)
CPD-1e	Corundum	55.12	1.38 (9)	1.41 (10)	1.66 (10)	1.66 (11)	1.52 (11)	1.55 (12)	1.76 (12)	1.79 (13)	2.88 (12)
	Fluorite	29.62	−0.73 (8)	−0.74 (8)	−1.05 (10)	−1.04 (10)	−0.84 (9)	−0.82 (9)	−1.14 (10)	−1.13 (11)	−2.49 (10)
	Zincite	15.25	−0.64 (5)	−0.66 (7)	−0.60 (5)	−0.61 (7)	−0.67 (6)	−0.72 (8)	−0.61 (6)	−0.66 (8)	−0.38 (7)
CPD-1f	Corundum	27.06	1.24 (12)	1.34 (12)	1.41 (12)	1.51 (12)	1.21 (19)	1.32 (19)	1.37 (19)	1.48 (19)	1.74 (19)
	Fluorite	17.72	−0.14 (7)	−0.05 (7)	−0.58 (10)	−0.51 (10)	−0.13 (8)	−0.04 (8)	−0.57 (10)	−0.50 (10)	−1.43 (10)
	Zincite	55.22	−1.10 (10)	−1.29 (11)	−0.83 (11)	−1.00 (12)	−1.08 (15)	−1.28 (16)	−0.80 (16)	−0.98 (16)	−0.30 (16)
CPD-1g	Corundum	31.37	1.26 (11)	1.29 (11)	1.43 (12)	1.46 (12)	1.75 (17)	1.81 (17)	1.92 (17)	1.98 (17)	2.64 (18)
	Fluorite	34.42	−0.60 (9)	−0.51 (9)	−0.91 (11)	−0.84 (11)	−0.83 (17)	−0.76 (11)	−1.16 (12)	−1.10 (13)	−2.55 (12)
	Zincite	34.21	−0.67 (8)	−0.78 (9)	−0.52 (9)	−0.62 (10)	−0.62 (11)	−1.05 (11)	−0.76 (11)	−0.88 (12)	−0.09 (12)
CPD-1h	Corundum	35.12	1.01 (11)	1.09 (11)	1.27 (12)	1.36 (12)	1.08 (16)	1.18 (16)	1.33 (16)	1.45 (16)	2.20 (17)
	Fluorite	34.69	−0.33 (9)	−0.19 (9)	−0.79 (11)	−0.67 (11)	−0.36 (11)	−0.24 (11)	−0.83 (12)	−0.72 (12)	−2.19 (12)
	Zincite	30.19	−0.69 (8)	−0.89 (9)	−0.48 (8)	−0.69 (9)	−0.71 (9)	−0.94 (10)	−0.50 (10)	−0.73 (11)	−0.01 (11)

**Table:** 

			MSP using hkl_Is							MSP using xo_Is		
Sample ID	Weighed wt%		R R f	R f R	R f f	f R R	f R f	f f R	f f f	R R x	R x R	x R R
CPD-1a	Corundum	1.15	0.11 (7)	0.18 (7)	0.21 (8)	0.49 (17)	0.49 (17)	0.61 (18)	0.62 (18)	0.07 (8)	0.15 (8)	0.81 (19)
	Fluorite	94.81	0.36 (9)	−0.25 (8)	−0.01 (10)	−0.26 (17)	−0.00 (17)	−0.68 (18)	−0.41 (19)	0.39 (10)	−0.26 (9)	−0.61 (18)
	Zincite	4.04	−0.47 (7)	0.07 (4)	−0.20 (7)	−0.23 (4)	−0.49 (7)	0.07 (4)	−0.21 (7)	−0.46 (7)	0.11 (5)	−0.21 (4)
CPD-1b	Corundum	94.31	−0.03 (6)	0.56 (6)	0.42 (7)	−0.13 (4)	−0.25 (6)	0.33 (6)	0.24 (8)	−0.02 (6)	0.56 (6)	−0.19 (5)
	Fluorite	4.33	−0.12 (4)	−0.59 (5)	−0.58 (5)	0.04 (4)	0.05 (4)	−0.44 (5)	−0.44 (6)	−0.12 (4)	−0.59 (5)	0.09 (4)
	Zincite	1.36	0.15 (5)	0.03 (2)	0.15 (5)	0.09 (2)	0.20 (5)	0.10 (2)	0.21 (5)	0.14 (5)	0.03 (2)	0.09 (2)
CPD-1c	Corundum	5.04	0.95 (11)	0.80 (11)	0.93 (11)	2.1 (4)	2.5 (4)	2.1 (4)	2.5 (4)	1.03 (11)	0.81 (11)	1.7 (4)
	Fluorite	1.36	0.09 (4)	0.31 (10)	0.40 (10)	0.02 (4)	0.10 (4)	0.29 (9)	0.37 (10)	0.10 (4)	0.31 (10)	0.03 (4)
	Zincite	93.59	−1.03 (12)	−1.10 (14)	−1.32 (14)	−2.1 (4)	−2.6 (4)	−2.4 (4)	−2.8 (4)	−1.12 (12)	−1.11 (14)	−1.7 (3)
CPD-1d	Corundum	13.53	1.33 (11)	1.68 (12)	2.00 (12)	0.32 (19)	0.6 (2)	0.9 (2)	1.2 (2)	1.33 (11)	1.70 (12)	0.2 (2)
	Fluorite	53.58	0.43 (11)	−2.67 (12)	−1.65 (12)	−0.16 (14)	0.90 (15)	−2.24 (15)	−1.18 (16)	0.44 (11)	−2.72 (12)	−0.10 (15)
	Zincite	32.89	−1.76 (10)	0.99 (9)	−0.35 (11)	−0.16 (10)	−1.50 (11)	1.30 (11)	−0.06 (12)	−1.77 (10)	1.02 (9)	−0.14 (11)
CPD-1e	Corundum	55.12	1.84 (10)	2.71 (10)	3.16 (11)	0.26 (11)	0.72 (12)	1.56 (12)	2.04 (13)	1.85 (10)	2.78 (10)	0.49 (13)
	Fluorite	29.62	−0.52 (8)	−2.38 (9)	−2.15 (10)	−0.01 (9)	0.25 (9)	−1.66 (10)	−1.42 (11)	−0.49 (8)	−2.46 (9)	−0.11 (10)
	Zincite	15.25	−1.31 (7)	−0.32 (5)	−1.00 (7)	−0.25 (6)	−0.96 (7)	0.11 (6)	−0.62 (8)	−1.35 (7)	−0.31 (5)	−0.36 (6)
CPD-1f	Corundum	27.06	2.15 (12)	1.72 (12)	2.66 (12)	0.19 (18)	1.09 (19)	0.65 (19)	1.58 (19)	2.15 (12)	1.72 (12)	2.29 (4)
	Fluorite	17.72	0.45 (8)	−1.49 (9)	−0.95 (9)	0.12 (8)	0.73 (8)	−1.25 (10)	0.70 (10)	0.45 (8)	−1.49 (9)	−0.38 (7)
	Zincite	55.22	−2.60 (11)	−0.23 (11)	−1.71 (12)	−0.31 (15)	−1.82 (16)	0.60 (16)	−0.88 (16)	−2.60 (11)	−0.23 (11)	−1.91 (6)
CPD-1g	Corundum	31.37	1.86 (12)	2.15 (12)	2.78 (12)	0.63 (17)	1.25 (17)	1.50 (17)	2.16 (17)	1.87 (12)	2.12 (12)	0.94 (18)
	Fluorite	34.42	−0.08 (10)	−2.36 (10)	−1.71 (11)	−0.27 (11)	0.41 (11)	−2.05 (12)	−1.41 (13)	0.08 (10)	−2.36 (10)	−0.35 (12)
	Zincite	34.21	−1.94 (9)	0.22 (9)	−1.07 (10)	−0.36 (10)	−1.66 (11)	0.55 (11)	−0.75 (12)	−1.94 (9)	0.24 (9)	−0.49 (11)
CPD-1h	Corundum	35.12	1.64 (11)	2.08 (12)	2.76 (12)	−0.10 (16)	0.55 (16)	0.94 (16)	1.65 (16)	1.68 (12)	2.15 (12)	0.37 (18)
	Fluorite	34.69	0.33 (9)	−2.25 (10)	−1.62 (11)	0.27 (11)	0.94 (11)	−1.68 (12)	−1.03 (12)	0.34 (10)	−2.34 (11)	0.04 (12)
	Zincite	30.19	−1.97 (9)	0.18 (8)	−1.13 (9)	−0.17 (9)	−1.49 (10)	0.73 (10)	−0.61 (11)	−2.01 (9)	0.20 (9)	−0.41 (10)

**Table 3:** Example of quantifying any component phase according to the chemical composition of the whole mixture sample, using the MSP method

	Mixture of cpd-1h		Corundum	Fluorite	Zincite
Chemical formula	(Al_0.693_Ca_0.439_Zn_0.369_O_1.409_F_0.878_)_*n*_		Al_2_O_3_	CaF_2_	ZnO
Molecular weight *M_k_*	*M_k_*/mol_f2 = 0.083		101.9613	78.0748	81.4084
MSP mol_f2			825.609	832.917	1443.86
Sum of LP-corrected intensity *S_k_*	2004.9	= sum of	522.199	586.681	896.02
*M_k_S_k_*/mol_f2	166.4067	≃ sum of	64.4907	54.9935	50.5198
		wt%	37.93%	32.35%	29.72%

## Data Availability

IUCr CPD round robin data are available at https://www.iucr.org/__data/iucr/powder/QARR/intro.htm. All other data and models supporting the results reported in this article are published in the supporting material.

## Footnotes

1 Equivalent forms of equation (12) have also been reported elsewhere (Hettich, 1935 ▸; Sayre, 1951 ▸; Kartha, 1953 ▸).

2 The *TOPAS*Stack macro containing the stack keyword was used to randomly shift the layer atoms flagged by the layer keyword in Cartesian **x**, **y**, **z** directions (stacking vector) in a supercell of 20 times the length of the *c* axis. The generated stacking layers have occupancies set to 0. The model is essentially a single-layer model in a supercell. However, the generated stacking layer atoms have geometric constraints set to avoid bumping into each other or into non-layer atoms, therefore keeping the model geometrically reasonable. With the keyword of continue_after_convergence, multiple refinements were executed before the result with the lowest *R*_wp_ was saved for the user.

3 For the purpose of reloading, *TOPAS* replaces the quotation marks with ‘§’, and replaces the divide mark ‘/’ with ‘£’ in the .pro file. Users need to replace them back once the .pro file has been loaded into *TOPAS*.

## Appendix A Meanings and relationships of the reserved intensity parameters in *TOPAS*

The *TOPAS* v7 software defines various reserved parameters concerning diffraction intensity for *hkl* reflections, including numerical_area, I_no_scale_pks, Iobs_no_scale_pks, I_after_scale_pks, I*etc*. Their meanings are summarized as below and are illustrated through a refinement of the corundum.raw data publicly available from the IUCr CPD QPA round robin (https://www.iucr.org/__data/iucr/powder/QARR/intro.htm).

(1) numerical_area returns a value of direct integration (counts × degrees) for the observed diffraction peak or diffraction pattern over the fitted 2θ range. This is similar to the ‘net area’ (cps × degrees) parameter in the *DIFFRAC.EVA* software calculated from the measured XRD pattern.

(2) I_no_scale_pks equals the product of ‘scale factor’, ‘multiplicity’ and squared structure factor |*F*|^2^ for each *hkl* reflection in the str structure model. This parameter does not contain the LP factor and therefore reflects the nature of the phase itself.

(3) Iobs_no_scale_pks partitions the measured intensity *Y*_obs_ at each 2θ step into the contributing phases, according to the ratio of their intensity contribution at that 2θ step. This parameter has the LP factor removed from the measured intensities. Therefore, when a good fit is achieved, this value should be close to the I_no_scale_pks value. This parameter is equivalent to the I parameter in hkl_Is, xo_Is and d_Is phases.

(4) I_after_scale_pks is equal to the product of I_no_scale_pks and all the scale_pks terms, including the LP factor. When a good fit is achieved, the value of I_after_scale_pks should be close to the numerical_area value for the same fitted 2θ range.

The I and numerical_area for each corundum diffraction peak extracted using the *TOPAS* file extract_numerical_area.inp (available in the supporting information) and the other three intensity parameters I_no_scale_pks, Iobs_no_scale_pks and I_after_scale_pks extracted using the *TOPAS* file extract_Is.inp (available in the supporting information) are compared in Fig. 11 ▸. It is obvious that the LP factor defined in TOPAS.inc forms the ratio between any group 1 parameter (numerical_area, I_after_scale_pks) and any group 2 parameter (I_no_scale_pks, Iobs_no_scale_pks, I) in hkl_Is/xo_Is phases.

where  represent the 2θ angle for the monochromator;  is the Bragg angle of the *hkl* reflection.

Therefore, it is clear that the numerical_area value in *TOPAS* is not equivalent to the value *S_k_* = ∑*_j_** I_jk_ G_jk_* in DDM, because numerical_area does not have the LP factor, which is defined as  by Toraya (2016 ▸), removed. DDM macros based on the numerical_area parameter are not equivalent to implementations of the direct derivation method (Toraya, 2016 ▸). The sum of I values in the *TOPAS*xo_Is or hkl_Is models should be used to calculate the *S_k_* value in DDM.
