# Supplementary material for: Incorporating the direct derivation method and molecular scattering power method into the Rietveld quantitative phase analysis routine in TOPAS
Source: J Appl Crystallogr. 2025 Jun 16;58(Pt 4):1159–73. doi: 10.1107/S1600576725004054 (PMC12321032; doi:10.1107/S1600576725004054)
Supplement: Supplementary file 1 [file j-58-01159-sup1.pdf]

JOURNAL OF
APPLIED
CRYSTALLOGRAPHY

Volume 58 (2025)

Supporting information for article:

Incorporating direct derivation method and molecular scattering power method into Rietveld quantitative phase analysis routine in TOPAS software

Xiaodong Wang and Henry Spratt

Supporting information

S1. TOPAS “mol_f2_Al2O3.inp” file to calculate molecular scattering power of Al_2O_3

This example calculates the molecular scattering power of Al_2O_3, without using any corundum crystal structure information. The molecule Al_2O_3 containing 5 atoms (3 “O” atoms and 2 “Al” atoms); therefore 5 imaginary crystals (3 identical “O” crystals and 2 identical “Al” crystal) of any arbitrary unit cell (for simplicity, a cubic cell of lattice parameter a = 10 Å is used in this example) are constructed, each of which contains a single constituent atom, either Al or O, at its eight corners. Below TOPAS INP file instructs the program to run 3 times. The 1st and 2nd runs export the square of structure factor for each reflection of the Al imaginary crystal and the O imaginary crystal into a Al.inc file and a O.inc file, respectively. The 3rd run sums up all the structure factor square saved in these .inc files separately, then calculate their sum according to the Al, O stichometry of Al_2O_3 in the dummy cell. The Al_2O_3 molecule scattering power is finally derived from above sum divided by the dummy cell volume and save to a file “mol_f2_Al2O3.inc” for the subsequent QPA INP to call. With this template, readers are encouraged to verify by themselves that the final value of molecule scattering power does not change much on different dummy unit cell sizes.

```
num_runs 3                'number of atoms in molecule + 1

macro Cal_f2_Lamda {
yobs_eqn !aac.xy = 1; min 5 max 150 del 0.01  'User defines 2Theta range
CuKa1(0.0001)             'User chooses radiation wavelength
}

macro dummy_cell {Cubic(10)} 'Any arbitrary cell larger than atom volumes
prm !cellvolume = 10^3;       'Dummy cell volume

macro Export_f2 {
out Current_Atom##.inc append
Out_String("prm sum_f2_")
Out(Get(phase_name),"%s")
Out_String(" 0 \n")
      phase_out Current_Atom##.inc append
            load out_record out_fmt out_eqn
            { "\nexisting_prm sum_f2_%s" = Get(phase_name);
              " +=    %12.5f;" = I_no_scale_pks;          } }

#if (Run_Number == 0)
Cal_f2_Lamda
STR(1) 'Imaginary crystal: SG:P1
macro Current_Atom {Al} 'Atomic Symbol
dummy_cell
phase_name Current_Atom
site Al_1 occ Al+3 1 beq 0.2952 'User provides "occ" "beq" if known
Export_f2
#endif
```

```
#if (Run_Number == 1)
Cal_f2_Lamda
STR(1) 'Imaginary crystal: SG:P1
macro Current_Atom {O}  'Atomic Symbol
dummy_cell
phase_name Current_Atom
site O_1 occ O-2 1 beq 0.1641   'User provides "occ" "beq" if known
Export_f2
#endif

#if (Run_Number == 2) 'Sum up all structural factors and calculate mol_f2
#include Al.inc         'include the exported "atom".inc files in run #1,
#include O.inc          'and in run #2
prm mol_f2_Al2O3 =(2*sum_f2_Al +3*sum_f2_O)/cellvolume; 'chemical formula
out mol_f2_Al2O3.inc
Out(mol_f2_Al2O3,"prm !mol_f2_Al2O3 = %15.3f\n")
#endif
```

S2. TOPAS "cal_C.inp" file to calculate C_k

An example of TOPAS file "cal_C.inp" file exporting C for fluorite is shown as below. It exports a "hkl.inc" file, which contains the C value in the last line for this phase. Information about macro AN used in this .inp file can be found in S3.

```
num_runs 2
#if (Run_Number == 0)
yobs_eqn !aac.xy = 1; min 1 max 150 del 0.01 'User defines 2Theta range
CuKa1(0.0001)
'---------------User inputs crystal structure-----------------
STR(225)
Cubic(5.4620)
phase_name "Fluorite"
site Ca_1 num_posns 1 x 0 y 0 z 0 occ Ca+2 1 beq 0.4856 local Ca_n2 =
Get(num_posns)*Get(occ)*(AN("Ca"))^2;
site F_1 num_posns 1 x 0.25 y 0.25 z 0.25 occ F-1 1 beq 0.720 local F_n2 =
Get(num_posns)*Get(occ)*(AN("F"))^2;
prm total_n2 = Ca_n2 + F_n2;
'---------------Export structural factors for each HKL---------
out hkl.inc append
Out_String("prm sum_F2 0 \n")
Out(Get(cell_volume)*(total_n2),"prm Toraya2016Eq8_Denominator %12.5f\n")
phase_out hkl.inc append
load out_record out_fmt out_eqn
{"\nexisting_prm sum_F2 +=    %12.5f;" = I_no_scale_pks;}
#endif
'------Sum up all structural factors and calculate C---------
#if (Run_Number == 1)
#include hkl.inc
out C_Fluorite_150.inc append 'Ck calculated for 2Th_UL = 150 deg
Out(sum_F2/Toraya2016Eq8_Denominator,"prm C_Fluorite = %10.3f;\n")
#endif
```

S3. TOPAS macros AN and AW

TOPAS macros "AN" and "AW" return atomic number (equals to electron number) and atomic weight for symbol of an element symbol respectively are shown below in two columns. The atomic weight data were copied from the "Standard Atomic Weight" column in the "isotopes.txt" file in the TOPAS installation folder. The atomic weight of some heavy atoms after No.109 are from www.periodictable.one. In order to use these macros, please paste them into the "local.inc" file in the TOPAS installation folder, before restarting TOPAS.

```
macro AN {Atomic_Number}
macro Atomic_Number(Atom) {
#if (Atom == "H")  1     #elseif
    (Atom == "He") 2     #elseif
    (Atom == "Li") 3     #elseif
    (Atom == "Be") 4     #elseif
    (Atom == "B")  5     #elseif
    (Atom == "C")  6     #elseif
    (Atom == "N")  7     #elseif
    (Atom == "O")  8     #elseif
    (Atom == "F")  9     #elseif
    (Atom == "Ne") 10    #elseif
    (Atom == "Na") 11    #elseif
    (Atom == "Mg") 12    #elseif
    (Atom == "Al") 13    #elseif
    (Atom == "Si") 14    #elseif
    (Atom == "P")  15    #elseif
    (Atom == "S")  16    #elseif
    (Atom == "Cl") 17    #elseif
    (Atom == "Ar") 18    #elseif
    (Atom == "K")  19    #elseif
    (Atom == "Ca") 20    #elseif
    (Atom == "Sc") 21    #elseif
    (Atom == "Ti") 22    #elseif
    (Atom == "V")  23    #elseif
    (Atom == "Cr") 24    #elseif
    (Atom == "Mn") 25    #elseif
    (Atom == "Fe") 26    #elseif
    (Atom == "Co") 27    #elseif
    (Atom == "Ni") 28    #elseif
    (Atom == "Cu") 29    #elseif
    (Atom == "Zn") 30    #elseif
    (Atom == "Ga") 31    #elseif
    (Atom == "Ge") 32    #elseif
    (Atom == "As") 33    #elseif
    (Atom == "Se") 34    #elseif
    (Atom == "Br") 35    #elseif
    (Atom == "Kr") 36    #elseif
    (Atom == "Rb") 37    #elseif
    (Atom == "Sr") 38    #elseif
    (Atom == "Y")  39    #elseif
    (Atom == "Zr") 40    #elseif
    (Atom == "Nb") 41    #elseif
    (Atom == "Mo") 42    #elseif
    (Atom == "Tc") 43    #elseif
    (Atom == "Ru") 44    #elseif
    (Atom == "Rh") 45    #elseif
    (Atom == "Pd") 46    #elseif
    (Atom == "Ag") 47    #elseif
    (Atom == "Cd") 48    #elseif
    (Atom == "In") 49    #elseif
    (Atom == "Sn") 50    #elseif
    (Atom == "Sb") 51    #elseif
    (Atom == "Te") 52    #elseif
    (Atom == "I")  53    #elseif
    (Atom == "Xe") 54    #elseif
    (Atom == "Cs") 55    #elseif
    (Atom == "Ba") 56    #elseif
    (Atom == "La") 57    #elseif
    (Atom == "Ce") 58    #elseif
    (Atom == "Pr") 59    #elseif
    (Atom == "Nd") 60    #elseif
    (Atom == "Pm") 61    #elseif
    (Atom == "Sm") 62    #elseif
    (Atom == "Eu") 63    #elseif
    (Atom == "Gd") 64    #elseif
    (Atom == "Tb") 65    #elseif
    (Atom == "Dy") 66    #elseif
    (Atom == "Ho") 67    #elseif
    (Atom == "Er") 68    #elseif
    (Atom == "Tm") 69    #elseif
    (Atom == "Yb") 70    #elseif
    (Atom == "Lu") 71    #elseif
    (Atom == "Hf") 72    #elseif
    (Atom == "Ta") 73    #elseif
    (Atom == "W")  74    #elseif
    (Atom == "Re") 75    #elseif
    (Atom == "Os") 76    #elseif
    (Atom == "Ir") 77    #elseif
    (Atom == "Pt") 78    #elseif
    (Atom == "Au") 79    #elseif
    (Atom == "Hg") 80    #elseif
```

```
    (Atom == "Tl") 81     #elseif
    (Atom == "Pb") 82     #elseif
    (Atom == "Bi") 83     #elseif
    (Atom == "Po") 84     #elseif
    (Atom == "At") 85     #elseif
    (Atom == "Rn") 86     #elseif
    (Atom == "Fr") 87     #elseif
    (Atom == "Ra") 88     #elseif
    (Atom == "Ac") 89     #elseif
    (Atom == "Th") 90     #elseif
    (Atom == "Pa") 91     #elseif
    (Atom == "U")  92     #elseif
    (Atom == "Np") 93     #elseif
    (Atom == "Pu") 94     #elseif
    (Atom == "Am") 95     #elseif
    (Atom == "Cm") 96     #elseif
    (Atom == "Bk") 97     #elseif
    (Atom == "Cf") 98     #elseif
    (Atom == "Es") 99     #elseif
    (Atom == "Fm") 100    #elseif
    (Atom == "Md") 101    #elseif
    (Atom == "No") 102    #elseif
    (Atom == "Lr") 103    #elseif
    (Atom == "Rf") 104    #elseif
    (Atom == "Db") 105    #elseif
    (Atom == "Sg") 106    #elseif
    (Atom == "Bh") 107    #elseif
    (Atom == "Hs") 108    #elseif
    (Atom == "Mt") 109    #elseif
    (Atom == "Ds") 110    #elseif
    (Atom == "Rg") 111    #elseif
    (Atom == "Cn") 112    #elseif
    (Atom == "Nh") 113    #elseif
    (Atom == "Fl") 114    #elseif
    (Atom == "Mc") 115    #elseif
    (Atom == "Lv") 116    #elseif
    (Atom == "Ts") 117    #elseif
    (Atom == "Og") 118    #endif
}

macro AW {Atomic_Weight}
macro Atomic_Weight(Atom) {
#if
(Atom == "H") 1.00794   #elseif
(Atom == "He") 4.002602 #elseif
(Atom == "Li") 6.941    #elseif
(Atom == "Be") 9.012182 #elseif
(Atom == "B") 10.811    #elseif
(Atom == "C") 12.0107   #elseif
(Atom == "N") 14.0067   #elseif
(Atom == "O") 15.9994   #elseif
(Atom == "F") 18.998403 #elseif
(Atom == "Ne") 20.1797  #elseif
(Atom == "Na") 22.98977 #elseif
(Atom == "Mg") 24.3050  #elseif
(Atom == "Al") 26.98154 #elseif
(Atom == "Si") 28.0855  #elseif
(Atom == "P") 30.973761 #elseif
(Atom == "S") 32.065    #elseif
(Atom == "Cl") 35.453   #elseif
(Atom == "Ar") 39.948   #elseif
(Atom == "K") 39.0983   #elseif
(Atom == "Ca") 40.078   #elseif
(Atom == "Sc") 44.95591 #elseif
(Atom == "Ti") 47.867   #elseif
(Atom == "V") 50.9415   #elseif
(Atom == "Cr") 51.9961  #elseif
(Atom == "Mn") 54.93805 #elseif
(Atom == "Fe") 55.845   #elseif
(Atom == "Co") 58.93320 #elseif
(Atom == "Ni") 58.6934  #elseif
(Atom == "Cu") 63.546   #elseif
(Atom == "Zn") 65.409   #elseif
(Atom == "Ga") 69.723   #elseif
(Atom == "Ge") 72.64    #elseif
(Atom == "As") 74.92160 #elseif
(Atom == "Se") 78.96    #elseif
(Atom == "Br") 79.904   #elseif
(Atom == "Kr") 83.798   #elseif
(Atom == "Rb") 85.4678  #elseif
(Atom == "Sr") 87.62    #elseif
(Atom == "Y") 88.90585  #elseif
(Atom == "Zr") 91.224   #elseif
(Atom == "Nb") 92.90638 #elseif
(Atom == "Mo") 95.94    #elseif
(Atom == "Tc") 98       #elseif
(Atom == "Ru") 101.07   #elseif
(Atom == "Rh") 102.9055 #elseif
(Atom == "Pd") 106.42   #elseif
(Atom == "Ag") 107.8682 #elseif
(Atom == "Cd") 112.411  #elseif
(Atom == "In") 114.818  #elseif
(Atom == "Sn") 118.710  #elseif
(Atom == "Sb") 121.760  #elseif
(Atom == "Te") 127.60   #elseif
(Atom == "I") 126.90447 #elseif
(Atom == "Xe") 131.293  #elseif
(Atom == "Cs") 132.9055 #elseif
(Atom == "Ba") 137.327  #elseif
(Atom == "La") 138.9055 #elseif
(Atom == "Ce") 140.116  #elseif
(Atom == "Pr") 140.907  #elseif
(Atom == "Nd") 144.24   #elseif
(Atom == "Pm") 145      #elseif
(Atom == "Sm") 150.36   #elseif
(Atom == "Eu") 151.964  #elseif
(Atom == "Gd") 157.25   #elseif
(Atom == "Tb") 158.9253 #elseif
```

```
(Atom == "Dy") 162.500  #elseif
(Atom == "Ho") 164.9303 #elseif
(Atom == "Er") 167.259  #elseif
(Atom == "Tm") 168.9342 #elseif
(Atom == "Yb") 173.04   #elseif
(Atom == "Lu") 174.967  #elseif
(Atom == "Hf") 178.49   #elseif
(Atom == "Ta") 180.9479 #elseif
(Atom == "W") 183.84    #elseif
(Atom == "Re") 186.207  #elseif
(Atom == "Os") 190.23   #elseif
(Atom == "Ir") 192.217  #elseif
(Atom == "Pt") 195.078  #elseif
(Atom == "Au") 196.9666 #elseif
(Atom == "Hg") 200.59   #elseif
(Atom == "Tl") 204.3833 #elseif
(Atom == "Pb") 207.2    #elseif
(Atom == "Bi") 208.9804 #elseif
(Atom == "Po") 209      #elseif
(Atom == "At") 210      #elseif
(Atom == "Rn") 222      #elseif
(Atom == "Fr") 223      #elseif
(Atom == "Ra") 226      #elseif
(Atom == "Ac") 227      #elseif
(Atom == "Th") 232.0381 #elseif
(Atom == "Pa") 231.0359 #elseif
(Atom == "U") 238.02891 #elseif
(Atom == "Np") 237      #elseif
(Atom == "Pu") 244      #elseif
(Atom == "Am") 243      #elseif
(Atom == "Cm") 247      #elseif
(Atom == "Bk") 247      #elseif
(Atom == "Cf") 251      #elseif
(Atom == "Es") 252      #elseif
(Atom == "Fm") 257      #elseif
(Atom == "Md") 258      #elseif
(Atom == "No") 259      #elseif
(Atom == "Lr") 262      #elseif
(Atom == "Rf") 261      #elseif
(Atom == "Db") 262      #elseif
(Atom == "Sg") 266      #elseif
(Atom == "Bh") 264      #elseif
(Atom == "Hs") 277      #elseif
(Atom == "Mt") 268      #elseif
(Atom == "Ds") 281      #elseif
(Atom == "Rg") 272      #elseif
(Atom == "Cn") 285      #elseif
(Atom == "Nh") 286      #elseif
(Atom == "Fl") 289      #elseif
(Atom == "Mc") 289      #elseif
(Atom == "Lv") 293      #elseif
(Atom == "Ts") 294      #elseif
(Atom == "Og") 294      #endif
}
```

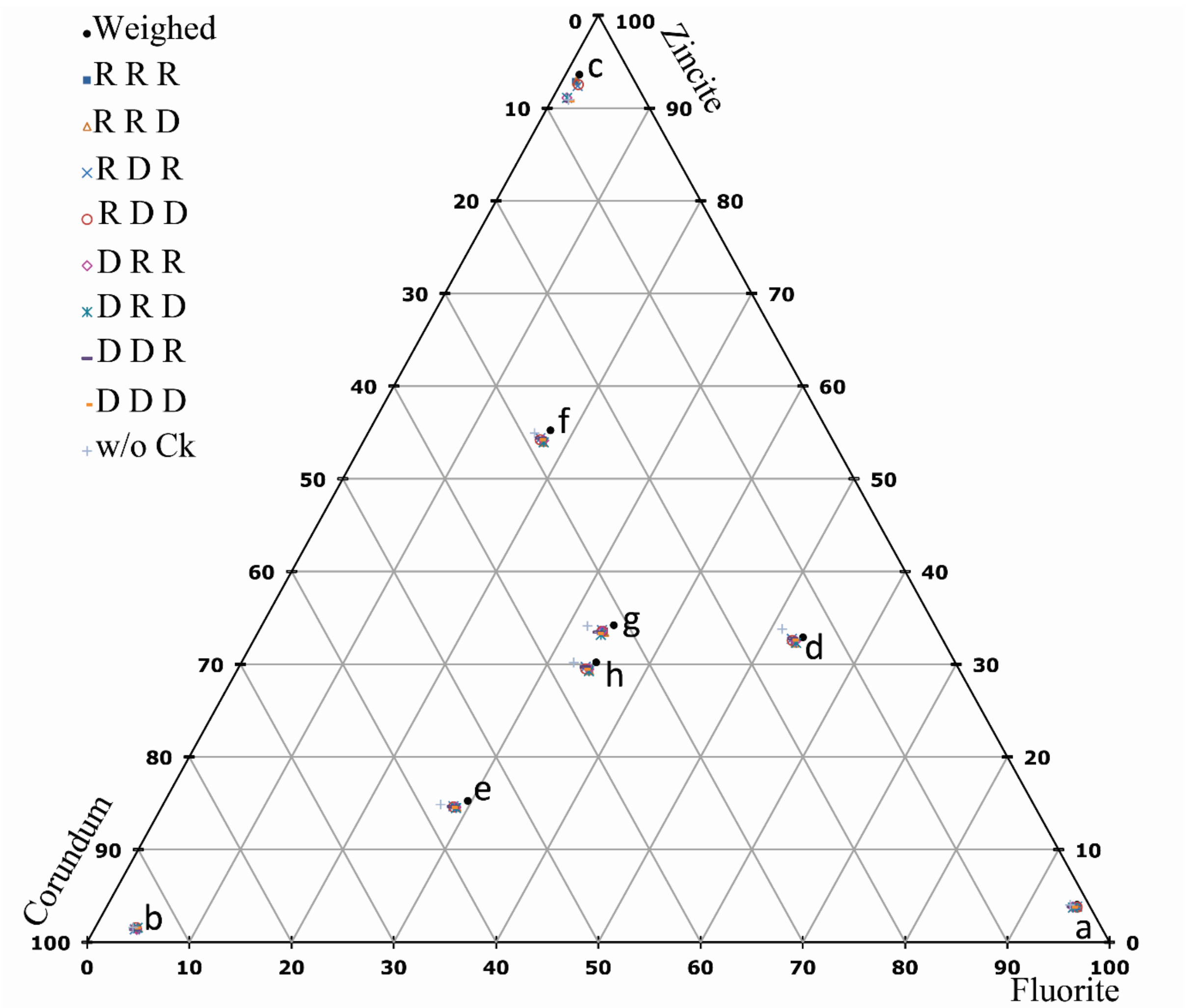

Figure S1 Discrepancies of QPA results using all possible combinations of "C_k corrected DDM" calculation and Rietveld QPA for the CPD-1 series dataset in the IUCr CPD round robin. The letters in legends, *e.g.* "R R D", represent models used for corundum, fluorite, and zincite respectively, where "R" stands for Rietveld QPA using "*str*" models, "D" stands for "C_k corrected DDM" calculation using "*hkl_Is*" models. The last legend "w/o C_k" marks QPA results using original DDM without C_k correction.

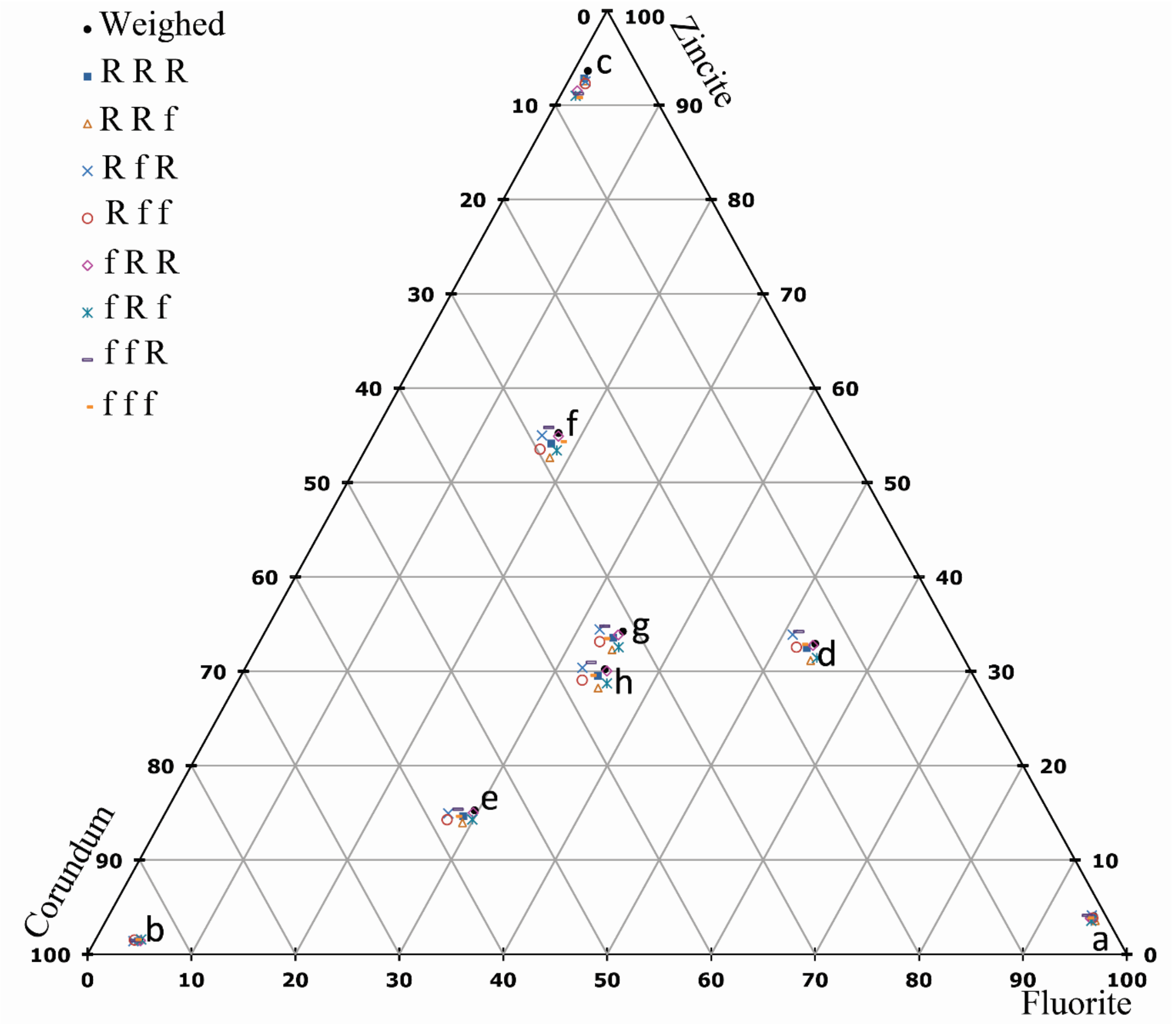

Figure S2 Discrepancies of QPA results using all possible combinations of MSP method (*hkl_Is* model) and Rietveld QPA for the CPD-1 series dataset in the IUCr CPD round robin. The letters in legends, *e.g.* “R R f”, represent models used for corundum, fluorite, and zincite respectively, where “R” stands for Rietveld QPA using “*str*” models, “f” stands for MSP method using “*hkl_Is*” models.

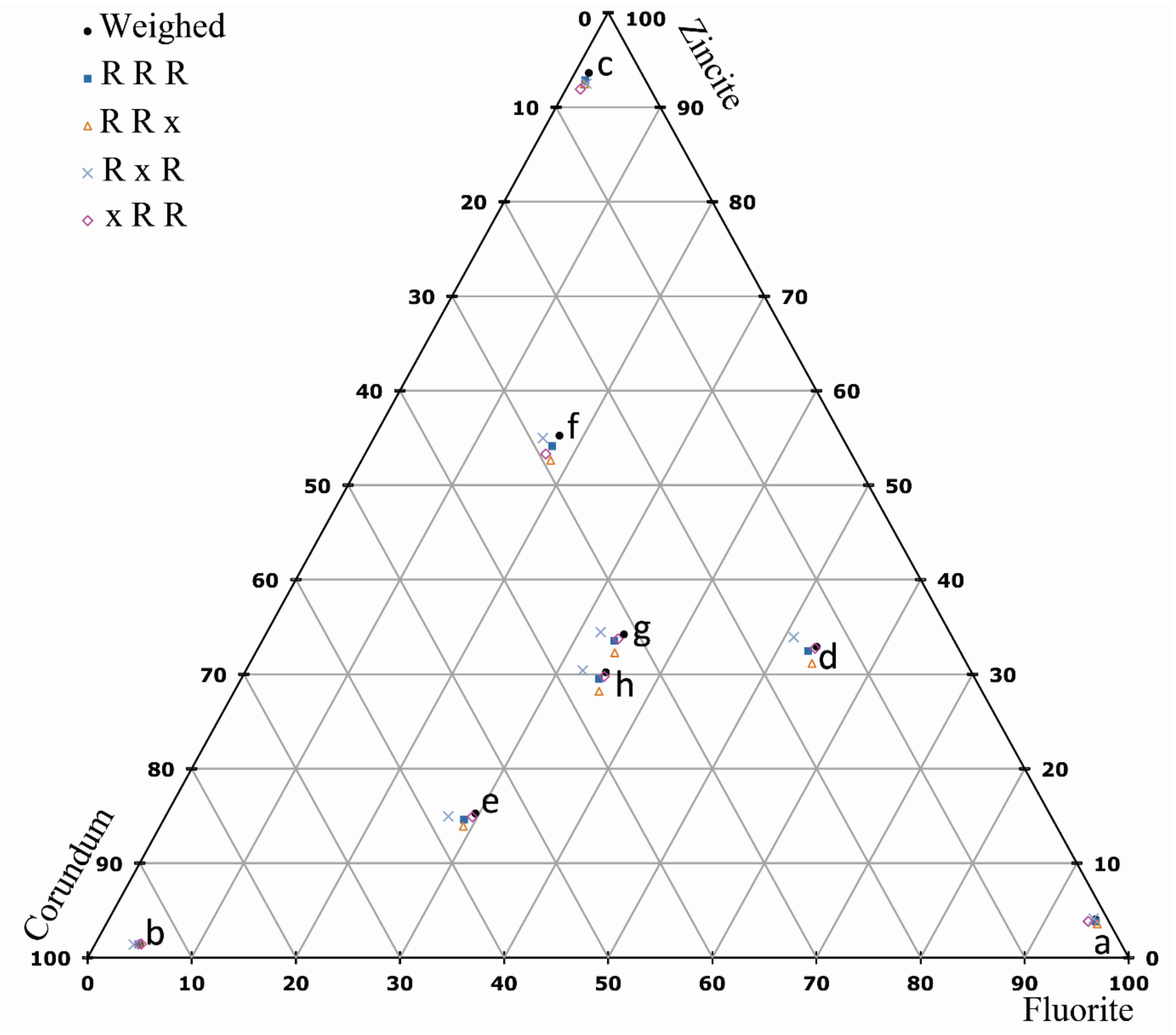

Figure S3 Discrepancies of QPA results using all possible combinations of MSP method (*xo_Is* model) and Rietveld QPA for the CPD-1 series dataset in the IUCr CPD round robin. The letters in legends, *e.g.* "R R x", represent models used for corundum, fluorite, and zincite respectively, where "R" stands for Rietveld QPA using "*str*" models, "x" stands for MSP method using "*xo_Is*" models.
